# Evolving therapy of adult acute lymphoblastic leukemia: state-of-the-art treatment and future directions

**DOI:** 10.1186/s13045-020-00905-2

**Published:** 2020-06-05

**Authors:** Bachar Samra, Elias Jabbour, Farhad Ravandi, Hagop Kantarjian, Nicholas J. Short

**Affiliations:** Department of Leukemia, The University of Texas MD Anderson Cancer Center, 1515 Holcombe Boulevard, Houston, TX 77030 USA

**Keywords:** Acute lymphoblastic leukemia, Monoclonal antibody, Inotuzumab ozogamicin, Blinatumomab, Chimeric antigen receptor

## Abstract

Recent years have witnessed major advances that have improved outcome of adults with acute lymphoblastic leukemia (ALL). The emergence of the concept of measurable residual disease has fine-tuned our prognostic models and guided our treatment decisions. The treatment paradigms of ALL have been revolutionized with the advent of tyrosine kinase inhibitors targeting BCR-ABL1, monoclonal antibodies targeting CD20 (rituximab), antibody-drug conjugates targeting CD22 (inotuzumab ozogamicin), bispecific antibodies (blinatumomab), and CD19 chimeric antigen receptor T cell therapy (tisagenlecleucel). These highly effective new agents are allowing for novel approaches that reduce reliance on intensive cytotoxic chemotherapy and hematopoietic stem cell transplantation in first remission. This comprehensive review will focus on the recent advances and future directions in novel therapeutic strategies in adult ALL.

## Introduction

Unlike pediatric acute lymphoblastic leukemia (ALL), which is curable in > 90% of cases, adult ALL has historically had a dismal prognosis, with limited treatment options and cure rates less than 40% [1, 2], due in part to higher-risk disease features in this population and significant chemotherapy-associated toxicity. B cell ALL accounts for approximately 75% of ALL cases and has historically been associated with inferior outcome compared with T cell ALL [2, 3]. Among B cell ALL cases, Philadelphia chromosome (Ph)-positive ALL was also historically associated with very poor outcomes in the pre-tyrosine kinase inhibitor (TKI) era [4–6]. However, in recent years, major advances in our understanding of the disease pathophysiology and genomics have led to better disease stratification and prognostication, leading to the identification of high-risk subgroups, such as Ph-like ALL and early T cell precursor (ETP) ALL. Furthermore, the detection and monitoring of measurable residual disease (MRD) has become a standard of care not only in stratifying patients but also in guiding treatment strategies [7, 8]. Excitingly, the therapeutic arsenal of ALL, particularly B cell ALL, has been markedly expanded with the advent of TKIs targeting the BCR-ABL1 tyrosine kinase, novel antibody constructs, and chimeric antigen receptor (CAR) T cell therapy [9–16]. By following the pediatric model of multiagent combination regimens, many clinical trials have been initiated over the past 5 years to investigate the best approaches to optimize these novel therapies for adult ALL, all of which may help to reduce our reliance on hematopoietic stem cell transplantation (HSCT) in first complete remission (CR1). This comprehensive review will focus on the recent advances and current standards in the therapy of different subsets of adult ALL. We also discuss novel strategies to combine or sequence these various treatment modalities in adults with the ultimate goal of recapitulating the success that has been made in the pediatric population.

## Philadelphia chromosome-negative ALL

Important lessons have been learned with the advent of monoclonal antibodies (moAb) targeting CD22 (inotuzumab ozogamicin [InO]) and CD19 (blinatumomab), which have dramatically improved outcome of adults with relapsed/refractory (R/R) B cell ALL. This has generated much interest in introducing the novel agents earlier during the course of therapy in order to deepen remissions, prevent relapses, and prolong survival. We will first highlight the available data on novel agents in the R/R setting where they were first studied, and then will discuss new combination/sequencing strategies that are being explored in the frontline setting.

### Relapsed/refractory setting

The prognosis of R/R ALL has historically been dismal with complete remission (CR) rates of 20-40%, median overall survival (OS) of 6 months, and cure rates of < 10% even with intensive salvage chemotherapy and HSCT [17, 18]. However, recent years have witnessed the introduction of novel agents, which showed significant survival benefit against standard therapies and expanded the armamentarium of ALL. The details of these single-agent studies have been reviewed extensively [19–21] and the data from pivotal trials are summarized in Table 1. We will therefore focus primarily on emerging therapies and the ways these novel agents are being explored in innovative combinations.

**Table 1:** Landmark trials of novel agents approved as single agents in B cell ALL

Drug	Study (phase)	Mechanism of action	Approved indication	Date of FDA approval	*N*	Median age, years [range]	CR/CRi rates, %	MRD negativity, %	Median RFS, months	Median OS, months	Reference
Inotuzomab ozogamicin	IN-NOVATE (III)	ADC anti-CD22	R/R B cell ALL	August 2017	109	47 [18-78]	81	78	5.0 (PFS)	7.7	[12]
Blinatumomab	TOWER (III)	BiTE anti CD3/CD19	R/R B cell ALL	September 2016	271	41 [18-80]	44	76	NR	7.7	[13]
Blinatumomab	ALCANTARA (II); Ph-positive ALL only	BiTE anti CD3/CD19	R/R B cell ALL	July 2017	45	55 [23-78]	36	88	6.7	7.1	[14]
Blinatumomab	BLAST (II)	BiTE anti CD3/CD19	MRD + ≥ 0.1% B cell ALL	March 2018	113	45 [18-76]	N/A	78	54% at 18 months	36.5	[15]
Tisagenlecleucel	ELIANA (II)	Anti-CD19 CAR T cells	CD19+ B cell ALL that is refractory or in second or later relapse in patients up to 25 years of age	August 2017	75	11 [3-23]	81	100	59% at 12 months	19.1	[16]

*B cell ALL* B cell acute lymphoblastic leukemia, *Ph* Philadelphia chromosome, *ADC* antibody drug conjugate, *BiTE*; bi-specific T cell engager, *R/R* relapsed refractory, *CR* complete remission, *MRD* measurable residual disease, *N* number of patients who received the novel agent, *CR* complete remission, *CRi* complete remission with incomplete hematologic recovery, *N/A* non applicable, *RFS* relapse-free survival, *PFS* progression-free survival, *EFS* event-free survival, *NR* not reached, *OS* overall survival

#### Inotuzimab ozogamicin monotherapy

InO is an anti-CD22 moAb conjugated to the cytotoxic antibiotic calicheamicin. Based on promising phase I/II data, InO was compared to standard salvage chemotherapy in a phase 3 multicenter trial (INO-VATE) of 218 adult patients with CD22+ B cell ALL [12, 22, 23]. The overall response and MRD negativity rates among responders were significantly higher with InO compared with chemotherapy (81% versus 29%, *P* < 0.001, and 78% versus 28%, *P* < 0.001, respectively). More patients who received InO were able to undergo HSCT (41% versus 11%; *P* < 0.001). The median remission duration and progression-free survival were significantly longer with InO (4.6 versus 3.1 months; *P* = 0.03, and 5.0 versus 1.8 months; *P* < 0.001, respectively). The median OS was 7.7 versus 6.7 months (*P* = 0.04). This was later confirmed with longer follow-up on 326 patients showing 2-year OS rates of 23% versus 10% (*P* = 0.01) in favor of InO [24]. Predictors for better survival included achievement of CR, MRD negativity, and consolidative HSCT. Patients who achieved MRD negativity derived more benefits regardless of the number of prior therapies [25]. InO was associated with more hepatotoxicity including veno-occlusive disease (VOD) but less hematologic and infectious complications compared with chemotherapy. VOD rate was 11% versus 1% with chemotherapy, mostly after HSCT and with use of dual-alkylator conditioning.

#### Blinatumomab monotherapy

Blinatumomab is a CD3/CD19 bispecific T cell engager moAb that has shown high efficacy in phase I/II studies in R/R B cell ALL, particularly in the setting of lower disease burden [26, 27]. The phase 3 multicenter international study TOWER subsequently showed superiority of blinatumomab compared to standard salvage chemotherapy in adult patients with heavily pre-treated R/R B cell ALL with higher CR rates (34% versus 16%; *P* < 0.001), MRD negativity (76% versus 48%), and longer median OS (7.7 versus 4 months; *P* = 0.001) [13]. The benefit was seen regardless of age, number of prior therapies, previous HSCT, or bone marrow blast percentage, but was more pronounced in first salvage (median OS 11.1 months versus 5.3 months). The two adverse events of interest were neurotoxicity and cytokine release syndrome (CRS), which were severe in 10% and 5% of cases, respectively.

#### Novel combination studies

The efficacy of these novel antibody constructs in ALL provides a rationale to combine either or both agents with lower intensity chemotherapy backbone with the goal of further improving outcomes. Table 2 summarizes the major novel combination trials in adult B cell ALL. Encouraging results have been shown with the combination of InO with mini-HCVD (which is a lower intensity version of the HCVAD regimen without doxorubicin) [34]. Among 59 patients treated, the CR or CR with incomplete hematological recovery (CRi) rate was 78%, and the MRD negativity rate was 82%. The median OS and relapse-free survival (RFS) were 11 months and 8 months, respectively. Almost half of the patients were able to undergo HSCT, in which case the median OS was 25 months. The incidence of VOD was 15%, mainly in patients with prior or subsequent HSCT. When these results were compared with historical controls treated with single-agent InO, there was significant improvement in outcomes (CR/CRi rates 75% versus 63%, *P* = 0.02, and median OS 9.3 months versus 5.6 months, *P* = 0.02). The study has now been amended to investigate the addition of 4 cycles of blinatumomab following 4 cycles of the combination InO and mini-HCVD [28]. This sequential strategy is potentially attractive as the addition of blinatumomab after debulking with mini-HCVD, and InO may lead to higher rates of MRD negativity and may also allow for the use of less chemotherapy and cumulative InO dose, ultimately reducing treatment-related mortality and improving long-term outcomes. In fact, the incidence of VOD was significantly reduced (from 15% to 5%) by using lower and fractionated dose of InO (first dose of 0.6 mg/m^2^ then 0.3 mg/m^2^ for each subsequent dose), and by spacing out the last dose of InO from HSCT by 3 to 6 months. In 62 patients treated in first salvage with mini-HCVD and InO, with or without blinatumomab, the CR/CRi and the 3-year OS rates were 92% and 42%, respectively. The 60-day mortality rate was 3% [35]. These results represent remarkable improvement considering the historical median OS in R/R B cell ALL is only 6 to 12 months. Longer follow-up is needed to assess the relative contribution of blinatumomab to the regimen.

**Table 2:** Published trials of combination of novel agents in adult Ph-negative ALL

Regimen	Patient population	*N*	Median age [range], years	Induction mortality, %	CR/CRi rate, %	MRD negativity, %	HSCT rate, %	CR duration, %	OS rate, %	Reference
**R/R Ph-negative ALL**										
Mini-HCVD + InO ± blinatumomab	Primary refractory 13% CR1 duration < 1 year 40% Prior HSCT 23%	84	35 [9-87]	2	80	80	40	52% (2-year)	39 (2-year)	[28]
CVP + InO (SWOG 1312)	Salvage 1: 44% Prior blinatumomab 38% Prior HSCT 19%	48	43 [20-79]	2	61	NR	30	NR	Median 10.9 months	[29]
Venetoclax + navitoclax	B cell ALL 50% T cell ALL 50% Median prior therapies: 4 Prior HSCT 14% Prior CAR T cells 17%	36	29 [6-72]	8	56	56	25	44% (6-month)	NR	[30]
**Frontline Ph-negative older ALL**										
Mini-HCVD + InO ± blinatumomab	Age ≥ 60 years	64	68 [60-81]	0	98	95	5	76% (3-year)	54 (3-year)	[31]
Blinatumomab + POMP (SWOG 1318)	Age > 60 years	31	73 [66-84]	0	66	92	3	DFS 56 (1-year)	65 (1-year)	[32]
**Frontline Ph-negative younger ALL**										
Sequential HCVAD + blinatumomab	Age < 60 years	27	38 [18-59]	0	100	96	30	RFS 76 (1-year)	89 (1-year)	[33]

*Ph* Philadelphia-chromosome; *ALL* acute lymphoblastic leukemia; *N* number, *CR1* first complete remission; *CR* complete remission; *CRi* complete remission with incomplete hematologic recovery; *MRD* measurable residual disease; *OS* overall survival; *EFS* event-free survival; *RFS* relapse-free survival; *mini-HCVD* mini-hyperfractionated cyclophosphamide, vincristine, dexamethasone; *InO* inotuzumab ozogamicin; *HSCT* hematopoietic stem cell transplant; *CAR* chimeric antigen receptor; *HCVAD* hyperfractionated cyclophosphamide, vincristine, Adriamycin, dexamethasone; *CVP* cyclophosphamide, vincristine, prednisone; *SWOG* South west oncology group; *POMP* prednisone, vincristine, methotrexate, mercaptopurine

#### Other drugs of interest

Preclinical studies have suggested that BCL-2 mRNA is highly expressed in multiple subtypes of ALL compared with normal pre-B controls, and that B-lineage ALL cells exhibit significant sensitivity to BCL-2 inhibition with venetoclax, resulting in rapid apoptotic cell death [36, 37]. Accordingly, the combination of venetoclax with lower-intensity chemotherapy is being evaluated in several prospective trials, including for untreated older patients (NCT03319901) or patients with R/R ALL (NCT03319901, NCT03504644, and NCT03808610). Navitoclax is another BH3 mimetic that inhibits BCL-2, BCL-XL, and BCL-W with encouraging antileukemic activity in ALL cells [38]. Preliminary results of a small phase 1 study evaluating the combination venetoclax and navitoclax in 36 patients with heavily pre-treated R/R ALL (including prior HSCT and CAR T cells) have shown a 50% CR/CRi rates, with 60% MRD negativity among responders [30].

Several moAbs targeting novel antigens, including CD25, CD123, and CD38, are in early development [39]. ADCT-402 is an anti-CD22 antibody-drug conjugate that delivers the cytotoxic agent tesirine (SG3249), which may have less hepatotoxicity than InO. It has shown safety and antileukemic activity in ALL and is now being investigated in a dose-expansion study (NCT02669264) [40]. Since high levels of regulatory T cells have been described as a mechanism of resistance to blinatumomab [41], immune checkpoint inhibitors are being investigated in combination with blinatumomab in an effort to restore T cell proliferation and improve outcomes (NCT03160079, NCT02879695).

#### Chimeric antigen receptor (CAR) T-cell therapy

CAR T cell therapy targeting CD19 is novel immunotherapy that has shown high clinical efficacy in R/R B cell ALL. Autologous T cells are genetically modified to express antibodies directed against CD19+ leukemic cells. There are currently 4 generations of CARs based on the type and number of co-stimulatory domains, which improve their expansion and persistence in vivo [42]. After lymphodepletion chemotherapy, T cells are infused into the patient in order to exert their direct cytotoxic effect and harness both innate and adapt immunity. The single infusion of tisagenlecleucel, a CD19 CAR T cell therapy, was evaluated in a pivotal phase 2 multicenter study of 75 children and young adults with R/R CD19+ B cell ALL, 61% of whom had received prior HSCT [16]. Among evaluable patients (80% of enrolled patients), the CR rate was 81%, all of which were MRD-negative. CAR T cells persisted for up to 20 months in the blood. The 18-month RFS and OS rates were 66% and 70%, respectively [43]. Adverse events of interest were CRS and neurotoxicity, occurring in 70% and 40%, respectively. Although, these toxicities are often of severe intensity, they are generally manageable with supportive therapy including the anti-interleukin 6 antibody, tocilizumab (only for CRS), and dexamethasone. This trial has led to the approval of tisagenlecleucel for R/R CD19+ B cell ALL after 2 prior lines of therapy or refractory to first-line therapy in patients up to 25 years of age.

Similar results were obtained with another CD19 CAR-T cell construct (containing CD28 and CD3 zeta chain co-stimulatory domains) from the Memorial Sloan Kettering Cancer Center in a phase 1 trial of 53 patients [44]. Notably, this trial included adult patients, and 15% were above 60 years of age. Higher tumor burden (bone marrow blasts > 5% or extramedullary disease) was associated with inferior outcomes (median OS 12 versus 20 months) and higher rates of CRS and neurotoxicity. The better efficacy and tolerability of CAR T cells in the context of low burden disease has generated interest in their wider use in patients with MRD-only disease as a potentially curative approach. A phase 3 trial is planned to compare tisagenlecleucel with blinatumomab or InO in adult patients with R/R B cell ALL (NCT03628053).

### Frontline therapy of older adults

Adults older than 60 years of age account for 20% of ALL cases and 50% of all ALL-related deaths across all age groups [45]. This striking difference between incidence and disease-specific mortality highlights the poor outcome and high unmet need of this older population. Survival rates have been historically dismal; less than 20% across many study groups primarily due to a higher risk of adverse-risk biology and comorbidities that may preclude intensive curative modalities (Table 3) [45–49]. Efforts made to modify intensive chemotherapy in order to decrease induction mortality and improve remission rates have resulted in sub-optimal success. For instance, in one retrospective study, despite dose reduction of cytarabine, the rates of induction mortality and death in CR in older patients treated with HCVAD regimen (hyper-fractionated cyclophosphamide, vincristine, doxorubicin, and dexamethasone alternating with high-dose methotrexate and cytarabine) were 10% and 35%, respectively [1, 46].

**Table 3:** Challenges in treating older patients with ALL

**Clinical factors**	
Decreased performance status	
Increased number of comorbidities	
Decreased organ function	
Polypharmacy	
Frequent dose reductions, delays, or omission	
Higher risk of adverse events (infections, neurotoxicity, secondary malignancies)	
**Biological factors**	
Increased incidence of adverse-risk karyotype (e.g., low hypodiploidy/near-triploidy, t(9;22), t(4;11), complex cytogenetics)	
Lower incidence of favorable-risk karyotype (hyperdiploidy, t(12;21), ETV6-RUNX1)	
Higher incidence of adverse risk molecular signatures (Philadelphia chromosome-like, TP53 mutation)	
**Social factors**	
Inadequate caregiver and/or social support	
Transportation/travel difficulties to tertiary centers	
**Other factors**	
Perceived lack of benefit of receiving anti-leukemia therapy rather than supportive/hospice care	

In the R/R setting, blinatumomab and InO have improved remission and survival rates irrespective of age (> 60 years and < 60 years) compared with standard salvage chemotherapies [12, 13]. Due to their acceptable toxicity profile and significant activity, there has been much interest in combining them with lower-intensity chemotherapy in the frontline setting in order to decrease toxicity and improve outcomes of older patients. For example, InO has been successfully combined with mini-HCVD with no induction mortality and with high clinical efficacy [50]. A similar combination of InO with lower intensity chemotherapy for older patients with Ph-negative B cell ALL is also being investigated in the EWALL-InO study (NCT03249870).

Similar to the R/R setting, the sequential combination of InO mini-HCVD followed by blinatumomab has been investigated in a phase 2 study in older untreated patients with promising results [31]. Among 64 patients treated with mini-HCVD and InO, with or without blinatumomab, the median age was 68 years (range 60–81 years) with 42% being older than 70 years. The CR rate was 98%, and the MRD negativity rate was 95%. The 3-year CR duration and OS rate were 76% and 54%, respectively. A propensity match score showed significant improvement compared to the historical 3-year OS rate of 32% with HCVAD in this older population (*P* = 0.007). Although no early death occurred in induction, the rate of death in remission was 33% and was significantly higher in those age ≥ 70 years compared to those age 60-69 years (50% versus 22%, respectively; *P* = 0.02). In order to mitigate the significant toxicity in this older population, the protocol has been amended to decrease the number of mini-HCVD plus InO cycles from four to two, and to replace POMP maintenance with blinatumomab monotherapy for patients ≥ 70 years of age.

The SWOG 1318 study evaluated chemotherapy-free induction and consolidation with blinatumomab (total of 4-5 cycles) followed by POMP maintenance (prednisone, vincristine, methotrexate, and 6-mercaptopurine). Thirty-one patients with a median age of 73 years (range 66-84) were treated. Early results showed no induction death, CR rate of 66% (among them 92% with negative MRD), and 1-year RFS and OS rates of 56% and 65%, respectively [32]. A planned phase 2 trial will investigate the combination of InO with blinatumomab in older untreated patients or R/R B cell ALL (NCT03739814).

### Frontline therapy of younger adults

In order to further improve outcomes of younger patients with newly diagnosed B cell ALL, a phase 2 trial is investigating the sequential use of HCVAD and blinatumomab with promising safety and efficacy [33]. The regimen consists of 4 cycles of HCVAD followed by 4 cycles of blinatumomab. Earlier incorporation of blinatumomab after 2 cycles of chemotherapy is allowed for patients at high risk for early relapse, particularly those with Ph-like ALL, complex karyotype, t(4;11), low-hypodiploidy/near triploidy, or persistent MRD. Four cycles of blinatumomab are also incorporated in the 12 cycles of POMP maintenance (each 3 cycles of POMP followed by 1 cycle of blinatumomab) for a total of 18 months of maintenance therapy. Among 27 patients treated (median age 27 years [range 18-57]), the CR and MRD negativity rates were 100% and 96%, respectively, with no induction death. One-third of patients underwent HSCT for high-risk features. With a median follow-up of 17 months, 93% are alive; one patient died after HSCT of a transplant-related complication, and one died of sepsis during re-induction after relapse. The 1-year RFS and OS rates were 76% and 89%, respectively. A randomized phase 3 trial is currently evaluating chemotherapy with or without blinatumomab for ALL in the frontline setting and may provide more definitive evidence about the benefit of early incorporation of blinatumomab (NCT02003222).

## Philadelphia chromosome-positive ALL

The Philadelphia chromosome, formed by reciprocal translocation t(9;22), is the most common chromosomal abnormality in adult ALL, with increasing incidence with age, reaching up to 50% in patients above 60 years of age [51, 52]. Historically, outcomes have been poor for patients with Ph-positive ALL with long term survival of less than 20% [4–6]. The addition of TKIs to chemotherapy has revolutionized therapy of patients with Ph-positive ALL, and is now standard of care. A summary of published frontline trials for Ph-positive ALL is provided in Table 4. The goal of therapy in Ph-positive ALL is not only to achieve and maintain CR, but to achieve complete molecular response (CMR) early in the treatment course. In one study, achievement of CMR within 3 months of treatment was the only independent prognostic factor for OS and identified patients who may have excellent long term survival without HSCT (4-year OS: 66%), thus potentially identifying patients in whom HSCT in CR1 may be safely deferred [66, 67]. The optimal duration of TKI therapy is not well-established but is often indefinite (in the absence of unacceptable toxicity) unless allogeneic HSCT is performed, after which most experts recommend post-HSCT TKI maintenance for approximately 1-2 years [68–71]. A small case series from the MD Anderson Cancer Center (MDACC) showed that TKI maintenance discontinuation outside of HSCT may be cautiously feasible in a subset of patients with deep and prolonged molecular remissions experiencing significant toxicity (e.g., CMR of least 5 years) [68]. In this retrospective analysis, 9 patients discontinued TKI maintenance (due to side effects), all of whom were in deep molecular response after a median of 70 months of therapy. After a median follow-up of 49 months, 3 molecular relapses occurred at a median of 6 months and the 4-year treatment-free remission rate was 65% (with all patients regaining molecular response after resuming TKI).

**Table 4:** Published frontline trials of TKI-based regimens in adult Ph-positive ALL

TKI	*N*	Median age, years [range]	CR rate, %	Induction mortality, %	Overall CMR rate, %	HSCT rate, %	RFS rate, %	OS rate, %	Reference
**Intensive chemotherapy + TKI**									
Imatinib	54	51 [17-84]	93	2	45	30	43 (5-year)	43 (5-year)	[10]
Imatinib	169	42 [16-64]	92	5	NR	72	50 (4-year)	38 (4-year)	[53]
Dasatinib	72	55 [21-80]	96	4	60	17	44 (5-year)	46 (5-year)	[54]
Nilotinib	90	47 [17-71]	91	9	86	70	72 (2-year)	72 (2-year)	[55]
Ponatinib	86	46 [21-80]	100	0	86	21	84 (3-year)	78 (3-year)	[56, 57]
**Lower-intensity chemotherapy + TKI**									
Imatinib	135	49 [18-59]	98	9	28	62	EFS 37 (5-year)	46 (5-year)	[11]
Dasatinib	71	69 [59-83]	96	4	24	10	EFS 28 (5-year)	36 (5-year)	[58]
Dasatinib	60	42 [19-60]	100	0	19	42	49 (3-year)	58 (3-year)	[59]
Nilotinib	79	65 [55-85]	94	2	58	16	42 (4-year)	47 (4-year)	[60]
Nilotinib	60	47 [18-59]	98	2	NR; MMR 80	52	85 (1-year)	96 (1-year)	[61]
**Steroids + TKI**									
Imatinib	30	69 [61-83]	100	0	4	NR	48 (1-year)	74 (1-year)	[62]
Dasatinib	53	54 [24-77]	100	0	15	34	51 (2-year)	69 (2-year)	[63]
Ponatinib	42	69 [27-85]	95	0	46	NR	NR	88 (1-year)	[64]
**Blinatumomab + TKI**									
Dasatinib	63	55 [24-82]	97	2	36	19	88 (1-year)	95 (1-year)	[65]

*TKI* tyrosine kinase inhibitor, *N* number, *CR* complete response, *CMR* complete molecular response, *NR* not reported, *MMR* major molecular response, *HSCT* allogeneic hematopoietic stem cell transplant, *RFS* relapse-free survival, *EFS* event-free survival, *OS* overall survival

### Intensive chemotherapy + TKI

The TKI era in Ph-positive ALL started when the addition of imatinib to intensive chemotherapy improved CR rates to ~ 95% and long term OS rates to 40-50%, which compared very favorably to the historical long term OS of < 10-20% in the pre-TKI era [9, 10, 53, 72, 73]. Dasatinib is a second-generation TKI that was combined with HCVAD regimen in two phase 2 trials, showing improvement upon imatinib data, with a CR rate of 96%, CMR rate of 56% and 3-year DFS and OS rates of 60-62% and 64-69%, respectively [9, 54, 74]. A landmark analysis of the SWOG study of HCVAD plus dasatinib in younger adults with Ph-positive ALL showed benefit for HSCT in CR1 in terms of RFS (*P* = 0.038) and OS (*P* = 0.037); however, MRD data were not available so it is not clear whether any subgroup preferentially benefited from consolidative HSCT. Nilotinib plus chemotherapy has also been studied with similarly promising results (2-year OS rate: 72%) [55].

*T315I* mutations of the *ABL1* kinase domain have been described in up to 75% of patients who relapse after treatment with first- or second-generation TKIs [58, 74]. This has led to interest in using ponatinib, a third-generation TKI with high potency and activity against this common resistance mutation [58, 74, 75]. The addition of ponatinib to HCVAD regimen has been tested in a phase 2 single-arm study with encouraging results. Initially, a ponatinib dose of 45 mg daily was used throughout the study. However, due to the increased incidence of severe vascular events, including 2 deaths related to ponatinib, the protocol was amended to reduce the dose of ponatinib to 30 mg daily after achievement of CR, and to 15 mg daily after achievement of CMR, with improved safety [56, 57, 76]. In the most recent update, 86 patients with a median age of 46 years have been treated [57]. The 3-month CMR rate was 74%, and the cumulative CMR rate was 84%. Only 18 patients (21%) underwent HSCT in CR1. With a median follow-up of 44 months, 71% of patients remain alive in remission, and only 3 relapses occurred while on ponatinib. The 5-year event-free survival (EFS) and OS rates were 68% and 74%, respectively. It is worth noting that while none of the TKIs have been compared head-to-head in Ph-positive ALL, one meta-analysis and one propensity-matched score analysis both showed superiority of ponatinib-based regimens over regimens containing earlier generation TKIs [77, 78]. Depth of remission, EFS, and OS rates all favored ponatinib.

### Lower-intensity chemotherapy + TKI

In order to decrease treatment-related toxicity, lower intensity regimens have been investigated in Ph-positive ALL, mainly in older patients who are unfit for intensive chemotherapy [11, 58, 60, 64]. Several single-arm trials have evaluated the combination of dasatinib or nilotinib with low-dose chemotherapy in older patients (> 55 years of age). In the EWALL-PH-01 trial, which used dasatinib, the CR rate was 96%, and the major molecular response (MMR) rate was 65% [58]. However, long-term outcomes were not optimal; the 5-year RFS and OS rates were only 28% and 36%, respectively. Additionally, a *T315I* mutation was present in most relapses (75%). Nilotinib yielded comparable results to dasatinib when combined with similar backbone chemotherapy in the EWAL-PH-02 trial [60]. The CR rate was 94%, and the 4-year RFS and OS were 42% and 47%, respectively.

Lower-intensity regimens are also being evaluated in younger patients, with the goal of reducing reliance on chemotherapy, and thus decreasing treatment-related toxicity. Notably, one randomized trial (GRAAPH-2005) compared the combination of imatinib with either HCVAD or lower-intensity version of the HCVAD (vincristine + prednisone in even cycles, thus, omitting doxorubicin and cyclophosphamide, while keeping methotrexate and cytarabine in odd cycles at standard dosing) in younger patients (median age 47 years). There was no statistically significant difference in 5-year EFS and OS rates between higher-intensity and lower-intensity regimens (42.2% versus 32.1%, *P* = 0.13, and 48.3%, versus 43.0%, *P* = 0.37, respectively) [11]. Importantly, this study showed that the combination of lower-intensity chemotherapy plus TKI may lead to similar long-term outcomes, and lower toxicity compared with intensive chemotherapy-based approach. However, there were a few limitations to the trial including the higher-than-expected 60-day mortality with HCVAD regimen (9%) and the intermittent dosing of imatinib (2 weeks on, 2 weeks off), which may not be optimal for continuous suppression of BCR-ABL1 [79]. The combination of dasatinib with prednisone was evaluated in younger patients in the GIMEMA LAL1509 trial (median age: 42 years). Patients who did not achieve CMR by day 85 (82% of the study population) subsequently received subsequent chemotherapy, with or without HSCT, whereas those who achieved CMR continued with dasatinib alone. Using this risk-adapted treatment approach, the 3-year OS rate was 58%. Those who achieved early CMR (18% of the cohort) and thus received no subsequent chemotherapy, had a very promising OS rate of 75% at 30 months. This suggests that early, deep response to lower-intensity therapy may allow for selection of patients who can have excellent outcomes with chemotherapy-free regimens. Similarly, in the phase II CALGB 10701 study, which combined dasatinib with prednisone in induction then added minimal chemotherapy in consolidation, high CR rates were seen (86%), allowing a third of patients to undergo allogeneic HSCT, among whom no relapses occurred at a median follow up of 23 months [80]. An ongoing phase 2 study at MDACC is investigating the combination of mini-HCVD with ponatinib and sequential blinatumomab in the frontline setting.

### Steroids + TKI

Several trials also evaluated the frontline combination of TKIs (most at a higher dose) with steroids in elderly frail patients with excellent CR rates and minimal toxicity [59, 62–64]. However, deep responses were, not unexpectedly, rarely attained, remissions were short, and relapses were common resulting in poor long-term survival. The rates of CMR appear to be higher with successive generations of TKIs (e.g., 46% with ponatinib, 18% with dasatinib, and 4% with imatinib) [59, 62–64]. Among 42 older patients treated with ponatinib and steroids (median age 68 [range 27-85]), the CMR and 1-year OS rates were 46% and 88%, respectively. Toxicity was high with 45 mg dosing and only 15/42 patients were still receiving this dose at week 24. One death was related to ponatinib [64]. While encouraging for this older population, novel lower-intensity strategies are needed to improve the CMR rate, as this has been shown to translate to superior long-term outcomes [67].

### Blinatumomab

Blinatumomab has shown safety and efficacy in heavily pre-treated R/R Ph-positive ALL in a single-arm multicenter phase 2 trial [14]. Among 45 patients treated (50% with prior exposure to ponatinib, 44% with prior HSCT, and 27% with *T315I* mutation), the CR/CRi rate was 36%, with 88% of responders achieving MRD negativity. Responses were observed regardless of *T315I* mutation status. Half of patients were able to undergo HSCT, and the median OS was 7.1 months.

The combination of blinatumomab with TKI (mainly ponatinib) has been shown to be safe and effective in a small case series of 15 patients from MDACC with 50% CR rate and 75% molecular response [81]. The GIMEMA group has recently presented early results from D-ALBA, the first trial investigating the sequential use of TKI/steroid (in induction) and blinatumomab (in consolidation) [65]. Sixty-three patients have been treated thus far with this regimen of prednisone, dasatinib, and blinatumomab. The CR rate was 98%, and the 1-year DFS rate was 88%. Deep molecular response increased throughout therapy (29% after induction, 60% after 2 cycles of blinatumomab, and 80% after 4 cycles). Notably, *T315I* mutation was noted in 6/15 patients with rising MRD in the induction phase, all of which were cleared after blinatumomab. Several similar trials are evaluating the combination of blinatumomab with dasatinib (NCT02143414, NCT04329325) and ponatinib (NCT03263572) in both frontline and R/R settings.

### Inotuzumab ozogamicin

The combination of InO with bosutinib is being evaluated in a phase 1/2 trial in R/R Ph-positive ALL (NCT02311998). Patients with *T315I* mutation are excluded. Early results have been presented on 14 patients with CR/CRi and CMR rates of 79% and 55%, respectively [82]. The median EFS and OS were 8.1 and 8.2 months, respectively. Although there is a theoretical concern for overlapping hepatic toxicity with the combination of InO and ponatinib, studies evaluating this combination are warranted given the high efficacy of both of these agents in ALL.

## Philadelphia-chromosome like ALL

Among B cell ALL, Ph-like ALL is a newly identified aggressive subtype that is characterized by a genomic signature similar to Ph-positive ALL, however, without the presence of *BCR-ABL1* rearrangement [83–85]. The incidence of Ph-like ranges from 15% in pediatric ALL to > 50% among young adults of Hispanic ethnicity [86]. Prognosis is poor with an estimated survival of < 30% [87]. Similar to Ph-positive ALL, *IKZF1* deletions are commonly found in Ph-like ALL (~ 70%) [85, 86]. More than half of patients have cytokine receptor-like factor 2 (CRLF2) rearrangement, among whom, 50% have concomitant activating mutations of Janus kinases (JAK1, JAK2, and JAK3). In patients without CRLF2 rearrangement/overexpression, genomic profiling may identify a variety of kinase-activating alterations, including rearrangements in *ABL* class genes (e.g., *ABL1*, *ABL2*, *CSF1R*, *PDGFRA*, and *PDGFRB*), *EPOR*, *JAK2*, and mutations involving *FLT3*, *IL7R*, or *SH2B3*, among others [88]. Adult patients with Ph-like ALL treated with conventional cytotoxic regimens, not only have approximately half the rate of MRD negativity, but their outcomes remain poor even when MRD negativity is achieved [89]. Whether the addition of novel agents (InO or blinatumomab) or HSCT is superior to intensive chemotherapy remains uncertain and this represents an area of active research. Notably, there may be a role for TKIs or other targeted therapies in a subset of patients with targetable fusions (e.g., dasatinib for *ABL* gene alterations; NCT02420717) [87, 90]. Given the prevalence of JAK/STAT alterations in Ph-like ALL, a few studies of ruxolitinib combination with chemotherapy are ongoing (NCT03117751, NCT02420717), although it is uncertain how beneficial this approach may be, as preclinical data suggests that lymphoblasts may not be dependent on continued activation of this pathway for maintenance of the malignant phenotype [91].

## T-cell ALL

T cell ALL is generally treated with the same chemotherapy regimens used for B-cell ALL with relatively similar response, except in ETP ALL, where response rates and outcomes are significantly worse [2]. The development of novel therapies for T cell ALL has lagged behind advancements seen in B cell ALL with no applicability of commercially available moAbs and CAR T cells, which may translate into inferior survival. Salvage options are limited, consisting mainly of conventional chemotherapy and HSCT among responders. Nelarabine is a T cell-specific purine analog that has shown efficacy in R/R T cell ALL (CR rates 30-40%), and has allowed some patients to undergo HSCT and achieve long-term survival [92–94]. This has warranted its exploration in the frontline setting in order to improve outcomes. In the pediatric experience, the addition of nelarabine to frontline Augmented Berlin-Frankfurt-Munster chemotherapy regimen (ABFM) in patients with T cell ALL up to 31 years of age improved the 4-year DFS rate from 83.3% with ABFM alone to 88.9% with the combination, *P* = 0.00332 [95, 96]. However, these results have not been replicated in adults yet. A single-arm phase 2 study from MDACC of nelarabine combined with frontline HCVAD regimen in 67 patients failed to improve CR duration or OS rates compared to historical controls treated with HCVAD alone [97]. This study has now been amended to include the incorporation of nelarabine, peg-aspragainase, and venetoclax into the HCVAD regimen. Additionally, the combination of nelarabine with standard intensive induction chemotherapy is being evaluated in a phase 2 randomized trial (UKALL14) in the frontline treatment of adults with T cell ALL.

ETP ALL is a distinct and aggressive subtype of T cell ALL characterized by CD1a(−), CD8(−), CD5(−/dim; < 75% expression), and positivity for one or more stem cell or myeloid antigens [98]. ETP ALL has been associated with lower frequency of *NOTCH1* mutation, lower response to therapy, higher rates of post-induction MRD positivity, and inferior survival compared with non-ETP ALL [89, 98–101]. Interestingly, ETP cells have been shown to be preferentially sensitive to the BCL-2 inhibitor, venetoclax [102]. The addition of venetoclax to lower-intensity chemotherapy in older adults with newly diagnosed ALL has yielded encouraging early results in interim results of 10 patients treated (3 with T cell ALL, including 2 with ETP ALL) with 90% CR/CRi and MRD negativity rate (for both) [103]. The combination of venetoclax and navitoclax may also be particularly promising in this subgroup.

## Special populations/considerations

### Adolescents and young adults (AYA)

According to the National Institute of Health, the AYA population is defined as patients between 16 and 39 years of age [104]. Several prospective trials that utilized pediatric-inspired intensive regimens in AYA patients have yielded CR rates in 85-90% and long-term EFS and OS in the 60-70% range [105–112]. Pediatric protocols employ extensive use of asparaginase, which can be associated with significant toxicity in adults (e.g., anaphylaxis, pancreatitis, hepatotoxicity, thrombosis, and coagulopathy), making them more challenging to deliver to older patients. The feasibility and efficacy of a pediatric-inspired regimen dedicated specifically for AYA patients has been recently demonstrated in a prospective multicenter study (CALGB 10403) [113]. The induction mortality was 3% and the CR rate was 89%. The 3-year EFS and OS rates were 59% and 73%, respectively, which are improved compared to historical controls (48% and 55%, respectively). The investigators at MDACC compared a pediatric-inspired regimen (ABFM, which contains daunorubicin, vincristine, asparaginase, steroids, cytarabine, and methotrexate) with the HCVAD regimen in AYA patients in a non-randomized study [114]. The 5-year OS rates were 60% in both groups. As expected, hepatic, pancreatic, and thrombotic toxicities were more common with ABFM, whereas myelosuppression and infectious complications were more common with HCVAD. Induction mortality was low in both groups (1%). These findings appear similar to the CALGB 10,403 results and support HCVAD as an acceptable regimen for AYA patients. In the absence of a randomized study comparing both regimens in the AYA population, it is important that practitioners adopt the protocol that matches their institution’s comfort level, and that they adhere to the protocol in its entirety, as these are key factors in achieving superior outcomes. More recently, the superior survival seen among patients with negative MRD has generated interest in integrating novel therapies early during induction in order to achieve deeper remission and improve cure rates. This is currently being investigated in the Alliance A041501 trial, which is adding InO to the CALGB 10403 backbone in AYA patients with newly diagnosed Ph-negative B cell ALL (NCT03150693).

### CD20-positive B-cell ALL

CD20 is a B cell marker that is expressed in 30-50% of precursor B cell ALL [115]. The addition of an anti-CD20 monoclonal antibody (moAb) to multiagent chemotherapy is a standard of care for younger patients (< 60 years old) with CD20+ B cell ALL (defined as CD20 expression ≥ 20%) [116–118]. This was first assessed in a prospective trial at MDACC where 12 doses of rituximab added to HCVAD improved outcome of patients who were younger than 60 years in terms of CR duration (67% versus 40%; *P* < 0.002) and OS rates (3-year OS 75% versus 47%; *P* = 0.003), compared with historical patients treated with HCVAD alone [118]. No benefit was observed in older patients, which was attributed to the high rate of myelosuppression-related deaths in this group. These results were later confirmed in the GRAALL-2005/R randomized study of adults younger than 60 years, which showed improvement in the 2-year EFS and OS rates from 52 to 65% (*P* = 0.038), and from 64 to 71% (*P* = 0.095; with censoring for HSCT, *P* = 0.018), respectively [117]. Of note, there was no increase in adverse events in patients receiving rituximab. Although there is no definitive evidence for the benefit of rituximab in older adults, it is reasonable to add it to frontline regimens in older adults given its manageable safety profile. In Burkitt leukemia/lymphoma, in which CD20 is universally and strongly expressed, the addition of rituximab to intensive chemotherapy backbone has improved survival and is a standard of care [116, 119, 120].

Ofatumumab is a second-generation anti-CD20 antibody with higher complement-dependent cytotoxicity and slower dissociation rate compared to rituximab [121]. Ofatumumab has been evaluated in a phase 2 study in combination with HCVAD in frontline B cell ALL with any level of CD20 expression (i.e., ≥ 1%) [122]. The clinical benefit was seen across all CD20+ subgroups (< 20% and > 20%). These results appear similar to historical cohorts treated with HCVAD + rituximab with 4-year EFS and OS rates of 59% and 68%, respectively. Therefore, ofatumumab is a reasonable option for CD20+ B cell ALL, especially with low level of CD20 expression—a population where rituximab has not been extensively studied. No clinical trials exist yet on obinutuzumab in ALL.

### Central nervous system (CNS) prophylaxis

Despite the relatively low incidence of CNS disease at presentation in ALL (5-10%), CNS relapses are common if no adequate CNS prophylaxis is given (~ 30% of patients in CR, and up to 75% in patients with R/R disease) [123–126]. Therefore, all current ALL regimens include CNS prophylaxis. The method of prophylaxis has varied according to the regimen being used, including intrathecal (IT) chemotherapy, high dose systemic therapy, radiation, or a combination of them. Efforts have been made to lower the doses or completely omit prophylactic cranial irradiation due to its significant cognitive toxicity, especially for long-term survivors. The combination of effective high-dose systemic therapy with CNS penetration (e.g., methotrexate or cytarabine) with IT chemotherapy has been equally effective, with CNS recurrence incidence of < 6%, similar to what is seen in regimens that used cranial radiation [127, 128]. Furthermore, one meta-analysis showed that cranial radiation in contemporary protocols was beneficial only in patients with overt CNS disease [129]. IT chemotherapy prophylaxis is typically given with alternating doses of methotrexate and cytarabine. The number of IT chemotherapy depends on the predetermined disease risk [128, 130]. For instance, the HCVAD regimen employs 8 IT chemotherapy doses for standard risk B or T cell ALL, 12 for Ph-positive ALL, and 16 for Burkitt leukemia, a risk-adapted approach that has resulted in a CNS recurrence rate < 4% [131].

## Incorporation of MRD into therapeutic decision-making

Despite high remission rates with frontline chemotherapy, unfortunately at least 40% of adults with ALL eventually relapse. This has been attributed mainly to the persistence of relatively chemoresistant MRD, which is low level of disease that is below the detection threshold of standard cyto-morphological assessment. MRD has refined risk stratification in ALL, as early clearance of MRD is reflective of high sensitivity to therapy and correlates with excellent long-term outcomes. Multiple retrospective and prospective studies have demonstrated that clearance of MRD at the end of induction or early consolidation is the strongest single prognostic factor in ALL, trumping all other predefined standard prognostic factors, such as white blood cells count and cytogenetics [8, 132–136]. This was confirmed in a large meta-analysis of more than 13,000 patients from 39 studies in both pediatric and adult populations [7]. However, although most patients who are MRD positive will eventually relapse, not all of them do, and, conversely, many relapses occur in MRD-negative patients. This highlights the limitations of current MRD testing, which primarily use multiparameter flow cytometry or polymerase chain reaction (PCR)-based strategies [137, 138]. Next-generation sequencing (NGS) and digital droplet PCR are other novel promising techniques with higher sensitivity (down to 10^−6^) that are being explored, but they are not standardized yet [139].

Consensus guidelines recommend MRD assessment to be done after induction, in early consolidation (after approximately 3 months of therapy), and every 3 months thereafter. MRD assessment should also be performed prior to HSCT [140]. Regardless of the method used (flow cytometry, PCR, NGS), a minimum sensitivity of 10^−4^ is recommended for adequate MRD assessment. In Ph-positive ALL, PCR for *BCR-ABL1* rearrangement is the preferred method of MRD monitoring. Although the optimal timing of MRD assessment is treatment-dependent, the earlier achievement of MRD (e.g., at end of induction) has generally been associated with better outcomes than MRD negativity achieved later over the course of therapy [138, 141]. MRD detection and monitoring has not only prognostic but also therapeutic implications. Patients with MRD positivity derive benefit from HSCT; however, the outcome of these patients post-HSCT remains suboptimal [135, 136, 142, 143]. In addition, blinatumomab has shown great efficacy in patients with MRD-positive disease. The phase 2 BLAST study treated 116 patients with blinatumomab who had persistent or recurrent MRD (detectable level of ≥ 0.1%) after chemotherapy. The MRD clearance rate was 80% after 2 cycles of blinatumomab [15]. The 4-year OS rate was 45%, which compares favorably to expectations of outcomes in MRD-positive patients (~ 30%) [7, 133, 144]. This has led to the approval of blinatumomab for this indication, the first such approval of an MRD-directed therapy [145]. However, new questions have emerged regarding optimal therapy following MRD clearance with blinatumomab such as the role of HSCT, or the relative benefit of TKI in MRD+ in Ph-positive ALL. The efficacy of InO for MRD-positive B cell ALL is also currently being evaluated in two clinical trials (NCT03610438 and NCT03441061).

## Evolving role of hematopoietic stem cell transplantation

Historically, nearly all patients with ALL were considered to have high relapse risk, and allogeneic HSCT was offered as consolidation for all fit candidates with suitable donors. Over the past two decades, the unprecedented progress in our understanding of disease biology and the improvement of frontline and salvage therapies have resulted in more accurate risk stratification, which is now primarily based on unique biological features (cytogenetics, genomic, and MRD status). This has allowed for better refinement of consolidative strategies in CR1, and thus HSCT is now largely reserved only for select patients with high-risk disease. Adverse risk cytogenetic features in adults include low hypodiploidy/near triploidy, t(4;11) [*KMT2A* rearrangement], complex karyotype (≥ 5 abnormalities), and Ph-like ALL all of which are indications for HSCT in CR1 [89, 146, 147]. Among T cell ALL, the ETP subtype and the lack of *NOTCH1* or *FBXW7* mutations are high-risk subgroups that may derive benefit from HSCT in CR1 [101, 148]. The presence of *NRAS/KRAS* mutations or *PTEN* gene alteration are other high-risk molecular features among T cell ALL [134]. However, there are no definitive data on the effect of HSCT in these subgroups [89, 149].

The advent of MRD assessment has refined the treatment landscape of ALL. Persistent MRD is generally considered an indication for HSCT in CR1 [106, 135, 136, 140, 146]. However, outcomes remain poor for patients with MRD positivity even when HSCT is performed. It is currently unclear whether patients who clear their MRD with blinatumomab or other novel agents would still derive benefit from HSCT. A post hoc analysis of the BLAST trial showed no difference in RFS or OS rates between patients who underwent HSCT after receiving blinatumomab and those who did not [15]. However, numbers were small and the equivalent survival outcome may be explained, at least partly, by the fact that HSCT-related mortality may offset the decreased relapse risk seen with HSCT. Furthermore, the role of consolidative HSCT after CAR T cell therapy remains controversial despite being favored by most experts, especially in HSCT-naive and fit patients [150].

In Ph-positive ALL, the added benefit of HSCT with the achievement of deep molecular remissions with more potent TKIs is now being questioned. We have previously shown that among patients treated with HCVAD plus a TKI without HSCT, the 4-year OS rate is 66% in patients who achieve CMR at 3 months, suggesting that HSCT may not be needed for those patients [67]. For example, the 5-year OS survival of patients treated with HCVAD plus ponatinib who did not undergo HSCT was 83% in the most recent update [57]. In contrast, patients who do not achieve at least MMR may benefit from HSCT in CR1 [68]. When imatinib was combined with HCVAD or a lower-intensity version of HCVAD in a randomized fashion, the benefit of HSCT was restricted to patients who did not achieve MMR after 2 cycles [11]. Taken together, these findings suggest that patients with Ph-positive ALL who achieve early deep molecular remissions may have excellent long term outcomes and may potentially be spared the toxicity of HSCT.

Moreover, the recent advance of haploidentical donor HSCT, has improved transplant-related outcomes for adults with ALL, especially older patients who are more likely to lack a matched donor [151–153]. This may be a particularly good option for patients with MRD-positive disease, as one prospective study showed better outcomes for haploidentical donor HSCT compared with matched sibling donor HSCT in this context [154]. In addition, the acceptable non-relapse mortality and favorable survival seen in patients older than 60 years treated with reduced-intensity conditioning (with post-HSCT OS up to 45% reported in this population) have made allogeneic HSCT a more feasible approach for older/less fit patients [155–157]. As both HSCT and non-HSCT options for ALL are rapidly evolving, decisions regarding indications for HSCT and proper patient selection are becoming increasingly complex.

## Conclusions and future directions

**Fig. 1:**
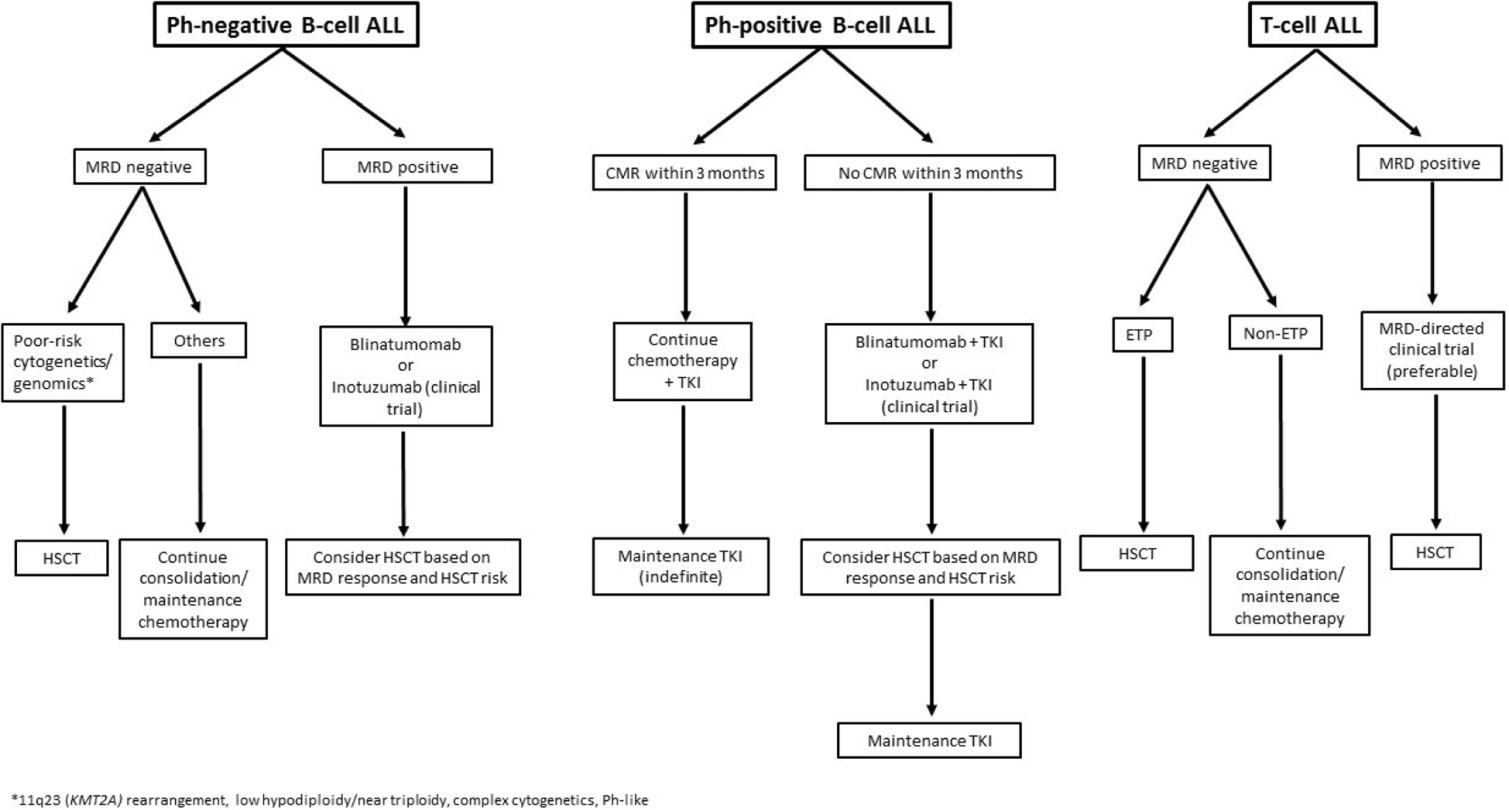
Proposed treatment algorithm of adult ALL according to MRD status. ALL, acute lymphoblastic leukemia; MRD, measurable residual disease; Ph, Philadelphia-chromosome; HSCT, hematopoietic stem cell transplant; CMR, complete molecular response; TKI, tyrosine kinase inhibitor; ETP, early T cell precursor

The outcome of adults with ALL remains suboptimal with cure rates of less than 60% in most subtypes. However, a better understanding of the disease biology has generated important knowledge on the prognostic and predictive value of MRD, which has helped guide our treatment strategies, such as intensification or referral to HSCT, the use of MRD-directed novel agents or even treatment de-escalation. We summarize in Fig. 1 our current MRD-based approach to treating ALL in adults. Undoubtedly this algorithm will continue to evolve.

Although the management of ALL is currently moving at an unprecedented pace, many challenges still remain. In Ph-positive ALL, with the dramatic improvement in outcomes by incorporating TKIs and the importance of monitoring MRD, the goal has become the achievement of early molecular remissions (MMR or, preferably, CMR within 3 months). A phase 3 randomized study is currently comparing reduced-intensity chemotherapy combination with either imatinib or ponatinib, which may help to clarify the optimal TKI to use in the frontline setting (NCT03589326). Whether using more potent TKIs such as ponatinib with minimal chemotherapy and/or blinatumomab may produce outcomes that are at least as good and less toxic than intensive chemotherapy-based regimens is yet to be determined. The combination of venetoclax with ponatinib and steroids is also being investigated in R/R Ph-positive ALL (NCT03576547).

With regards to CAR T cell therapy, more data in adult patients and strategies to improve availability of cells and their safety profile are needed. “Off-the-shelf, ready-to-use” allogeneic CAR T cells may overcome the logistical challenge of delivery time [158] and are currently being investigated (NCT02799550). Furthermore, despite the high rates of remission and MRD negativity, many responses are not durable and relapses occur in ~ 50%, including CD19-negative relapses. Due to the universal expression of CD22 in B cell ALL cells, new strategies to improve CAR T cell outcomes may include CAR-T cells directed against CD22 [159], and CD19/CD22 dual-targeted constructs [160, 161]. Most recently, allogeneic CD19 CAR-NK cells have shown high efficacy and minimal toxicity in R/R chronic lymphocytic leukemia and B cell lymphoma [162]. However, their role in the treatment of ALL is uncertain at the present time.

Unfortunately, there continue to be some high-risk subtypes of ALL such as Ph-like, *KMT2A*-rearrangement, and T cell ALL (particularly ETP ALL), in which progress has lagged behind and thus are in crucial need of novel therapeutic strategies. Based on pre-clinical evidence of high expression of CD38 in T cells, daratumumab, a human moAb against CD38 approved in multiple myeloma, has shown a significant anti-leukemic effect in small case series of R/R T cell ALL and Ph-positive ALL [163, 164] and is being evaluated in a phase 2 clinical trial (NCT03384654). CAR T cell products against T cell ALL are also in development, although their construction has been more challenging than for B cell ALL. Barriers include difficulty in harvesting an adequate number of autologous T cells and the fratricide (self-killing) effect due to shared antigens between CAR T cells, normal T cells, and leukemic T cells, such as CD7 which is present in ~ 95% of T cell ALL [165]. One emerging way to overcome these hurdles is CRISPR/Cas9 editing to delete CD7 from healthy “off-the-shelf” donor T cells while transducing them with CD7 targeting CAR. The early results of this approach are promising [166]. In an interim analysis of a phase I study of CD7 CAR T cells for R/R T cell ALL, all 5 patients treated (median age 24 years and median of 5 prior lines of therapy) achieved CR, with only 1 of them relapsing after a median follow-up of 3 months. No CRS or graft-versus disease was seen.

With the development of novel, effective therapies such as InO, blinatumomab, and CAR T cells, our treatment options have not only expanded, but our focus is shifting toward strategies that minimize cytotoxic chemotherapy and HSCT. However, until the best combination and sequence of these novel agents are fully defined, enrollment in clinical trials, and referral to tertiary centers remains crucial. With continued efforts to optimize the available therapies with novel combinations, there is reason for optimism that the treatment of adult ALL may eventually become another oncological success story.

## Data Availability

Not applicable as no datasets were generated or analyzed.

## Abbreviations

ALL: Acute lymphoblastic leukemia
AYA: Adolescent and young adult
Ph: Philadelphia chromosome
CR: Complete remission
CRi: Complete remission with incomplete count recovery
CR1: First complete remission
CMR: Complete molecular remission
R/R: Relapse refractory
MRD: Measurable residual disease
EFS: Event-free survival
RFS: Relapse-free survival
OS: Overall survival
HSCT: Hematopoietic stem cell transplant
TKI: Tyrosine kinase inhibitor
InO: Inotuzumab ozogamicin
moAb: Monoclonal antibody
CAR: Chimeric antigen receptor
ETP: Early T cell precursor
HCVAD: Hyper-fractionated cyclophosphamide, vincristine, adriamycin, and dexamethasone alternating with high-dose methotrexate and cytarabine
Mini-HCVD: Mini- hyper-fractionated cyclophosphamide, vincristine, and dexamethasone
MDACC: MD Anderson Cancer Center
CNS: Central nervous system
VOD: Veno-occlusive disease
CRS: Cytokine release syndrome
IT: Intrathecal

## References

[1] Kantarjian H, Thomas D, O’Brien S, Cortes J, Giles F, Jeha S (2004). Long-term follow-up results of hyperfractionated cyclophosphamide, vincristine, doxorubicin, and dexamethasone (Hyper-CVAD), a dose-intensive regimen, in adult acute lymphocytic leukemia. Cancer..

[2] Rowe JM, Buck G, Burnett AK, Chopra R, Wiernik PH, Richards SM (2005). Induction therapy for adults with acute lymphoblastic leukemia: results of more than 1500 patients from the international ALL trial: MRC UKALL XII/ECOG E2993. Blood..

[3] Marks DI, Paietta EM, Moorman AV, Richards SM, Buck G, DeWald G (2009). T-cell acute lymphoblastic leukemia in adults: clinical features, immunophenotype, cytogenetics, and outcome from the large randomized prospective trial (UKALL XII/ECOG 2993). Blood..

[4] Chao NJ, Blume KG, Forman SJ, Snyder DS (1995). Long-term follow-up of allogeneic bone marrow recipients for Philadelphia chromosome-positive acute lymphoblastic leukemia. Blood..

[5] Pui CH, Crist WM, Look AT (1990). Biology and clinical significance of cytogenetic abnormalities in childhood acute lymphoblastic leukemia. Blood..

[6] Thomas X, Thiebaut A, Olteanu N, Danaila C, Charrin C, Archimbaud E, et al. Philadelphia chromosome positive adult acute lymphoblastic leukemia: characteristics, prognostic factors and treatment outcome. Hematol Cell Ther. 1998;40(3):119–28. 9698220

[7] Berry DA, Zhou S, Higley H, Mukundan L, Fu S, Reaman GH, et al. Association of minimal residual disease with clinical outcome in pediatric and adult acute lymphoblastic leukemia: a meta-analysis. JAMA Oncol. 2017;3(7):e170580. 10.1001/jamaoncol.2017.0580 PMC5824235 28494052

[8] Ribera JM, Oriol A, Morgades M, Montesinos P, Sarra J, Gonzalez-Campos J, et al. Treatment of high-risk Philadelphia chromosome-negative acute lymphoblastic leukemia in adolescents and adults according to early cytologic response and minimal residual disease after consolidation assessed by flow cytometry: final results of the PETHEMA ALL-AR-03 trial. J Clin Oncol. 2014;32(15):1595–604. 10.1200/JCO.2013.52.2425 24752047

[9] Ravandi F, O'Brien S, Thomas D, Faderl S, Jones D, Garris R (2010). First report of phase 2 study of dasatinib with hyper-CVAD for the frontline treatment of patients with Philadelphia chromosome-positive (Ph+) acute lymphoblastic leukemia. Blood..

[10] Daver N, Thomas D, Ravandi F, Cortes J, Garris R, Jabbour E (2015). Final report of a phase II study of imatinib mesylate with hyper-CVAD for the front-line treatment of adult patients with Philadelphia chromosome-positive acute lymphoblastic leukemia. Haematologica..

[11] Chalandon Y, Thomas X, Hayette S, Cayuela JM, Abbal C, Huguet F (2015). Randomized study of reduced-intensity chemotherapy combined with imatinib in adults with Ph-positive acute lymphoblastic leukemia. Blood..

[12] Kantarjian HM, DeAngelo DJ, Stelljes M, Martinelli G, Liedtke M, Stock W (2016). Inotuzumab ozogamicin versus standard therapy for acute lymphoblastic leukemia. N Engl J Med.

[13] Kantarjian H, Stein A, Gokbuget N, Fielding AK, Schuh AC, Ribera JM (2017). Blinatumomab versus chemotherapy for advanced acute lymphoblastic leukemia. N Engl J Med.

[14] Martinelli G, Boissel N, Chevallier P, Ottmann O, Gökbuget N, Topp MS (2017). Complete hematologic and molecular response in adult patients with relapsed/refractory Philadelphia chromosome–positive B-precursor acute lymphoblastic leukemia following treatment with blinatumomab: results from a phase II, single-arm, multicenter study. J Clin Oncol.

[15] Gokbuget N, Dombret H, Bonifacio M, Reichle A, Graux C, Faul C (2018). Blinatumomab for minimal residual disease in adults with B-cell precursor acute lymphoblastic leukemia. Blood..

[16] Maude SL, Laetsch TW, Buechner J, Rives S, Boyer M, Bittencourt H (2018). Tisagenlecleucel in children and young adults with B-cell lymphoblastic leukemia. N Engl J Med.

[17] Gokbuget N, Dombret H, Ribera JM, Fielding AK, Advani A, Bassan R (2016). International reference analysis of outcomes in adults with B-precursor Ph-negative relapsed/refractory acute lymphoblastic leukemia. Haematologica..

[18] Fielding AK, Richards SM, Chopra R, Lazarus HM, Litzow MR, Buck G (2007). Outcome of 609 adults after relapse of acute lymphoblastic leukemia (ALL); an MRC UKALL12/ECOG 2993 study. Blood..

[19] Jabbour E, Pui CH, Kantarjian H. Progress and innovations in the management of adult acute lymphoblastic leukemia. JAMA Oncol. 2018;4(10):1413–20. 10.1001/jamaoncol.2018.1915 29931220

[20] Liu D, Zhao J, Song Y, Luo X, Yang T (2019). Clinical trial update on bispecific antibodies, antibody-drug conjugates, and antibody-containing regimens for acute lymphoblastic leukemia. J Hematol Oncol.

[21] Kantarjian H, Jabbour E. Incorporating immunotherapy into the treatment strategies of B-cell adult acute lymphoblastic leukemia: the role of blinatumomab and inotuzumab ozogamicin. Am Soc Clin Oncol Educ Book. 2018;38:574–8. 10.1200/EDBK_199505 30231308

[22] Kantarjian H, Thomas D, Jorgensen J, Kebriaei P, Jabbour E, Rytting M (2013). Results of inotuzumab ozogamicin, a CD22 monoclonal antibody, in refractory and relapsed acute lymphocytic leukemia. Cancer..

[23] DeAngelo DJ, Stock W, Stein AS, Shustov A, Liedtke M, Schiffer CA (2017). Inotuzumab ozogamicin in adults with relapsed or refractory CD22-positive acute lymphoblastic leukemia: a phase 1/2 study. Blood Adv.

[24] Kantarjian HM, DeAngelo DJ, Stelljes M, Liedtke M, Stock W, Gökbuget N (2019). Inotuzumab ozogamicin versus standard of care in relapsed or refractory acute lymphoblastic leukemia: final report and long-term survival follow-up from the randomized, phase 3 INO-VATE study. Cancer..

[25] Jabbour E, Gökbuget N, Advani AS, Stelljes M, Stock W, Liedtke M, et al. Impact of minimal residual disease (MRD) status in clinical outcomes of patients with relapsed/refractory (R/R) acute lymphoblastic leukemia (ALL) treated with inotuzumab ozogamicin (InO) in the phase 3 INO-VATE trial. J Clin Oncol. 2018;36(15_suppl):7013.

[26] Topp MS, Gokbuget N, Stein AS, Zugmaier G, O’Brien S, Bargou RC (2015). Safety and activity of blinatumomab for adult patients with relapsed or refractory B-precursor acute lymphoblastic leukaemia: a multicentre, single-arm, phase 2 study. The Lancet Oncology.

[27] Topp MS, Gokbuget N, Zugmaier G, Klappers P, Stelljes M, Neumann S, et al. Phase II trial of the anti-CD19 bispecific T cell-engager blinatumomab shows hematologic and molecular remissions in patients with relapsed or refractory B-precursor acute lymphoblastic leukemia. J Clin Oncol. 2014;32(36):4134–40. 10.1200/JCO.2014.56.3247 25385737

[28] Sasaki K, Kantarjian HM, Ravandi F, Short NJ, Kebriaei P, Huang X, et al. Sequential combination of low-intensity chemotherapy (mini-hyper-CVD) plus inotuzumab ozogamicin with or without blinatumomab in patients with relapsed/refractory Philadelphia chromosome-negative acute lymphoblastic leukemia (ALL): a phase 2 trial. Blood. 2018;132(Supplement 1):553.

[29] Advani AS, Moseley A, Liedtke M, O'Donnell MR, Aldoss I, Mims MP, et al. SWOG 1312 final results: a phase 1 trial of inotuzumab in combination with CVP (cyclophosphamide, vincristine, prednisone) for relapsed/refractory CD22+ acute leukemia. Blood. 2019;134(Supplement_1):227.

[30] Lacayo NJ, Pullarkat VA, Stock W, Jabbour E, Bajel A, Rubnitz J, et al. Safety and efficacy of venetoclax in combination with navitoclax in adult and pediatric relapsed/refractory acute lymphoblastic leukemia and lymphoblastic lymphoma. Blood. 2019;134(Supplement_1):285.

[31] Short NJ, Kantarjian HM, Ravandi F, Huang X, Jain N, Sasaki K, et al. Updated results of a phase II study of reduced-intensity chemotherapy with mini-hyper-CVD in combination with inotuzumab ozogamicin, with or without blinatumomab, in older adults with newly diagnosed Philadelphia chromosome-negative acute lymphoblastic leukemia. Blood. 2019;134(Supplement_1):823.

[32] Advani AS, Moseley A, O’Dwyer KM, Wood B, Fang M, Wieduwilt MJ, et al. Results of SWOG 1318: a phase 2 trial of blinatumomab followed by pomp (prednisone, vincristine, methotrexate, 6-mercaptopurine) maintenance in elderly patients with newly diagnosed Philadelphia chromosome negative B-cell acute lymphoblastic leukemia. Blood. 2018;132(Supplement 1):33.

[33] Richard-Carpentier G, Kantarjian HM, Short NJ, Ravandi F, Ferrajoli A, Schroeder HM, et al. Updated results from the phase II study of hyper-CVAD in sequential combination with blinatumomab in newly diagnosed adults with B-cell acute lymphoblastic leukemia (B-ALL). Blood. 2019;134(Supplement_1):3807.

[34] Jabbour E, Ravandi F, Kebriaei P, Huang X, Short NJ, Thomas D, et al. Salvage chemoimmunotherapy with inotuzumab ozogamicin combined with mini–hyper-CVD for patients with relapsed or refractory Philadelphia chromosome–negative acute lymphoblastic leukemia: a phase 2 clinical trial. JAMA Oncol. 2018;4(2):230–4. 10.1001/jamaoncol.2017.2380 PMC5838597 28859185

[35] Sasaki K, Kantarjian HM, Ravandi F, Short NJ, Kebriaei P, Huang X, et al. Sequential combination of inotuzumab ozogamicin (InO) with low-intensity chemotherapy (mini-hyper-CVD) with or without blinatumomab is highly effective in patients (pts) with Philadelphia chromosome-negative acute lymphoblastic leukemia (ALL) in first relapse. Blood. 2019;134(Supplement_1):3806.

[36] Geng H, Brennan S, Milne TA, Chen WY, Li Y, Hurtz C (2012). Integrative epigenomic analysis identifies biomarkers and therapeutic targets in adult B-acute lymphoblastic leukemia. Cancer Discov.

[37] Alford SE, Kothari A, Loeff FC, Eichhorn JM, Sakurikar N, Goselink HM (2015). BH3 Inhibitor sensitivity and Bcl-2 dependence in primary acute lymphoblastic leukemia cells. Cancer Res.

[38] Frismantas V, Dobay MP, Rinaldi A, Tchinda J, Dunn SH, Kunz J (2017). Ex vivo drug response profiling detects recurrent sensitivity patterns in drug-resistant acute lymphoblastic leukemia. Blood..

[39] Guerra VA, Jabbour EJ, Ravandi F, Kantarjian H, Short NJ. Novel monoclonal antibody-based treatment strategies in adults with acute lymphoblastic leukemia. Ther Adv Hematol. 2019;10:2040620719849496. 10.1177/2040620719849496 PMC6535741 31205644

[40] Jain N, Klisovic RB, Stock W, Ungar D, Zeidan AM, Atallah E, et al. Interim data from a phase 1 study evaluating pyrrolobenzodiazepine-based antibody drug conjugate ADCT-402 (loncastuximab tesirine) targeting CD19 for relapsed or refractory B-cell acute lymphoblastic leukemia. Blood. 2017;130(Supplement 1):1321.

[41] Duell J, Dittrich M, Bedke T, Mueller T, Eisele F, Rosenwald A (2017). Frequency of regulatory T cells determines the outcome of the T-cell-engaging antibody blinatumomab in patients with B-precursor ALL. Leukemia..

[42] Zhao J, Song Y, Liu D (2019). Clinical trials of dual-target CAR T cells, donor-derived CAR T cells, and universal CAR T cells for acute lymphoid leukemia. J Hematol Oncol.

[43] Grupp SA, Maude SL, Rives S, Baruchel A, Boyer MW, Bittencourt H, et al. Updated analysis of the efficacy and safety of tisagenlecleucel in pediatric and young adult patients with relapsed/refractory (r/r) acute lymphoblastic leukemia. Blood. 2018;132(Supplement 1):895.

[44] Park JH, Riviere I, Gonen M, Wang X, Senechal B, Curran KJ (2018). Long-term follow-up of CD19 CAR therapy in acute lymphoblastic leukemia. N Engl J Med.

[45] Short NJ, Kantarjian H, Jabbour E, Ravandi F. Novel therapies for older adults with acute lymphoblastic leukemia. Curr Hematol Malig Rep. 2018;13(2):91–9. 10.1007/s11899-018-0440-3 29423571

[46] O'Brien S, Thomas DA, Ravandi F, Faderl S, Pierce S, Kantarjian H (2008). Results of the hyperfractionated cyclophosphamide, vincristine, doxorubicin, and dexamethasone regimen in elderly patients with acute lymphocytic leukemia. Cancer..

[47] Gokbuget N (2013). How I treat older patients with ALL. Blood..

[48] Li S, Molony JT, Chia V, Katz AJ (2016). Patient characteristics and treatment patterns in elderly patients newly diagnosed with acute lymphoblastic leukemia (ALL) using 100% Medicare ALL data. Blood..

[49] Geyer MB, Hsu M, Devlin SM, Tallman MS, Douer D, Park JH (2017). Overall survival among older US adults with ALL remains low despite modest improvement since 1980: SEER analysis. Blood..

[50] Kantarjian H, Ravandi F, Short NJ, Huang X, Jain N, Sasaki K, et al. Inotuzumab ozogamicin in combination with low-intensity chemotherapy for older patients with Philadelphia chromosome-negative acute lymphoblastic leukaemia: a single-arm, phase 2 study. Lancet Oncol. 2018;19(2):240–8. 10.1016/S1470-2045(18)30011-1 PMC11537312 29352703

[51] Ribeiro RC, Abromowitch M, Raimondi SC, Murphy SB, Behm F, Williams DL (1987). Clinical and biologic hallmarks of the Philadelphia chromosome in childhood acute lymphoblastic leukemia. Blood..

[52] Moorman AV, Chilton L, Wilkinson J, Ensor HM, Bown N, Proctor SJ (2010). A population-based cytogenetic study of adults with acute lymphoblastic leukemia. Blood..

[53] Fielding AK, Rowe JM, Buck G, Foroni L, Gerrard G, Litzow MR (2014). UKALLXII/ECOG2993: addition of imatinib to a standard treatment regimen enhances long-term outcomes in Philadelphia positive acute lymphoblastic leukemia. Blood..

[54] Ravandi F, O'Brien SM, Cortes JE, Thomas DM, Garris R, Faderl S (2015). Long-term follow-up of a phase 2 study of chemotherapy plus dasatinib for the initial treatment of patients with Philadelphia chromosome-positive acute lymphoblastic leukemia. Cancer..

[55] Kim DY, Joo YD, Lim SN, Kim SD, Lee JH, Lee JH (2015). Nilotinib combined with multiagent chemotherapy for newly diagnosed Philadelphia-positive acute lymphoblastic leukemia. Blood..

[56] Jabbour E, Kantarjian H, Ravandi F, Thomas D, Huang X, Faderl S, et al. Combination of hyper-CVAD with ponatinib as first-line therapy for patients with Philadelphia chromosome-positive acute lymphoblastic leukaemia: a single-centre, phase 2 study. Lancet Oncol. 2015;16(15):1547–55. 10.1016/S1470-2045(15)00207-7 PMC4816046 26432046

[57] Short NJ, Kantarjian HM, Ravandi F, Huang X, Daver NG, DiNardo CD, et al. Long-term safety and efficacy of hyper-CVAD plus ponatinib as frontline therapy for adults with Philadelphia chromosome-positive acute lymphoblastic leukemia. Blood. 2019;134(Supplement_1):283.

[58] Rousselot P, Coude MM, Gokbuget N, Gambacorti Passerini C, Hayette S, Cayuela JM (2016). Dasatinib and low-intensity chemotherapy in elderly patients with Philadelphia chromosome-positive ALL. Blood..

[59] Chiaretti S, Vitale A, Elia L, Fedullo AL, Albino S, Piciocchi A (2015). Multicenter total therapy Gimema LAL 1509 protocol for de novo adult Ph + acute lymphoblastic leukemia (ALL) patients. Updated results and refined genetic-based prognostic stratification. Blood.

[60] Ottmann OG, Pfeifer H, Cayuela J-M, Spiekermann K, Jung W, Beck J, et al. Nilotinib (Tasigna®) and low intensity chemotherapy for first-line treatment of elderly patients with BCR-ABL1-positive acute lymphoblastic leukemia: final results of a prospective multicenter trial (EWALL-PH02). Blood. 2018;132(Supplement 1):31.

[61] Chalandon Y, Rousselot P, Cayuela J-M, Thomas X, Clappier E, Havelange V, et al. Nilotinib combined with lower-intensity chemotherapy for front-line treatment of younger adults with Ph-positive acute lymphoblastic leukemia: interim analysis of the GRAAPH-2014 trial. Eur Hematol Assoc. 2018;2(1):410.

[62] Vignetti M, Fazi P, Cimino G, Martinelli G, Di Raimondo F, Ferrara F (2007). Imatinib plus steroids induces complete remissions and prolonged survival in elderly Philadelphia chromosome-positive patients with acute lymphoblastic leukemia without additional chemotherapy: results of the Gruppo Italiano Malattie Ematologiche dell’Adulto (GIMEMA) LAL0201-B protocol. Blood..

[63] Foa R, Vitale A, Vignetti M, Meloni G, Guarini A, De Propris MS (2011). Dasatinib as first-line treatment for adult patients with Philadelphia chromosome-positive acute lymphoblastic leukemia. Blood..

[64] Martinelli G, Piciocchi A, Papayannidis C, Paolini S, Robustelli V, Soverini S, et al. First report of the Gimema LAL1811 phase II prospective study of the combination of steroids with ponatinib as frontline therapy of elderly or unfit patients with Philadelphia chromosome-positive acute lymphoblastic leukemia. Blood. 2017;130(Supplement 1):99.

[65] Chiaretti S, Bassan R, Vitale A, Elia L, Piciocchi A, Puzzolo C, et al. Dasatinib-blinatumomab combination for the front-line treatment of adult Ph + ALL patients. Updated results of the Gimema LAL2116 D-Alba trial. Blood. 2019;134(Supplement_1):740.

[66] Ravandi F, Jorgensen JL, O'Brien SM, Jabbour E, Thomas DA, Borthakur G (2016). Minimal residual disease assessed by multi-parameter flow cytometry is highly prognostic in adult patients with acute lymphoblastic leukaemia. Br J Haematol.

[67] Short NJ, Jabbour E, Sasaki K, Patel K, O’Brien SM, Cortes JE (2016). Impact of complete molecular response on survival in patients with Philadelphia chromosome-positive acute lymphoblastic leukemia. Blood..

[68] Samra B, Kantarjian HM, Sasaki K, Konopleva MY, Khouri R, O’Brien SM, et al. Discontinuation of tyrosine kinase inhibitors (TKIs) in Philadelphia chromosome-positive (Ph+) acute lymphoblastic leukemia (ALL). Blood. 2019;134(Supplement_1):3819-.

[69] Pfeifer H, Wassmann B, Bethge W, Dengler J, Bornhauser M, Stadler M (2013). Randomized comparison of prophylactic and minimal residual disease-triggered imatinib after allogeneic stem cell transplantation for BCR-ABL1-positive acute lymphoblastic leukemia. Leukemia..

[70] Carpenter PA, Snyder DS, Flowers ME, Sanders JE, Gooley TA, Martin PJ (2007). Prophylactic administration of imatinib after hematopoietic cell transplantation for high-risk Philadelphia chromosome-positive leukemia. Blood..

[71] Giebel S, Czyz A, Ottmann O, Baron F, Brissot E, Ciceri F (2016). Use of tyrosine kinase inhibitors to prevent relapse after allogeneic hematopoietic stem cell transplantation for patients with Philadelphia chromosome-positive acute lymphoblastic leukemia: a position statement of the acute leukemia working party of the European Society for Blood and Marrow Transplantation. Cancer..

[72] Tanguy-Schmidt A, Rousselot P, Chalandon Y, Cayuela JM, Hayette S, Vekemans MC, et al. Long-term follow-up of the imatinib GRAAPH-2003 study in newly diagnosed patients with de novo Philadelphia chromosome-positive acute lymphoblastic leukemia: a GRAALL study. Biology Blood Marrow Transplant. 2013;19(1):150–5. 10.1016/j.bbmt.2012.08.021 22960387

[73] Piccaluga PP, Paolini S, Martinelli G (2007). Tyrosine kinase inhibitors for the treatment of Philadelphia chromosome-positive adult acute lymphoblastic leukemia. Cancer..

[74] Ravandi F, Othus M, O’Brien SM, Forman SJ, Ha CS, Wong JYC (2016). US Intergroup study of chemotherapy plus dasatinib and allogeneic stem cell transplant in Philadelphia chromosome positive ALL. Blood Adv.

[75] Pfeifer H, Wassmann B, Pavlova A, Wunderle L, Oldenburg J, Binckebanck A (2007). Kinase domain mutations of BCR-ABL frequently precede imatinib-based therapy and give rise to relapse in patients with de novo Philadelphia-positive acute lymphoblastic leukemia (Ph + ALL). Blood..

[76] Short NJ, Kantarjian HM, Ravandi F, Daver NG, Pemmaraju N, Thomas DA, et al. Frontline hyper-CVAD plus ponatinib for patients with Philadelphia chromosome-positive acute lymphoblastic leukemia: updated results of a phase II study. J Clin Oncol. 2017;35(15_suppl):7013-.

[77] Sasaki K, Jabbour EJ, Ravandi F, Short NJ, Thomas DA, Garcia-Manero G (2016). Hyper-CVAD plus ponatinib versus hyper-CVAD plus dasatinib as frontline therapy for patients with Philadelphia chromosome-positive acute lymphoblastic leukemia: a propensity score analysis. Cancer..

[78] Jabbour E, DerSarkissian M, Duh MS, McCormick N, Cheng WY, McGarry LJ, et al. Efficacy of ponatinib versus earlier generation tyrosine kinase inhibitors for front-line treatment of newly diagnosed Philadelphia-positive acute lymphoblastic leukemia. Clin Lymphoma Myeloma Leuk. 2018;18(4):257–65. 10.1016/j.clml.2018.02.010 29519619

[79] Biondi A, Schrappe M, De Lorenzo P, Castor A, Lucchini G, Gandemer V, et al. Imatinib after induction for treatment of children and adolescents with Philadelphia-chromosome-positive acute lymphoblastic leukaemia (EsPhALL): a randomised, open-label, intergroup study. Lancet Oncol. 2012;13(9):936–45. 10.1016/S1470-2045(12)70377-7 PMC3431502 22898679

[80] Wieduwilt MJ, Yin J, Wetzler M, Uy GL, Powell BL, Kolitz JE (2016). A phase II study of dasatinib and dexamethasone as primary therapy followed by hematopoietic cell transplantation for adults with Philadelphia chromosome-positive acute lymphoblastic leukemia: CALGB study 10701 (Alliance). Blood.

[81] Assi R, Kantarjian H, Short NJ, Daver N, Takahashi K, Garcia-Manero G, et al. Safety and efficacy of blinatumomab in combination with a tyrosine kinase inhibitor for the treatment of relapsed Philadelphia chromosome-positive leukemia. Clin Lymphoma Myeloma Leuk. 2017;17(12):897–901. 10.1016/j.clml.2017.08.101 28927784

[82] Jain N, Cortes JE, Ravandi F, Konopleva M, Alvarado Y, Kadia T, et al. Inotuzumab ozogamicin in combination with bosutinib for patients with relapsed or refractory Ph + ALL or CML in lymphoid blast phase. Blood. 2017;130(Supplement 1):143.

[83] Den Boer ML, van Slegtenhorst M, De Menezes RX, Cheok MH, Buijs-Gladdines JG, Peters ST, et al. A subtype of childhood acute lymphoblastic leukaemia with poor treatment outcome: a genome-wide classification study. Lancet Oncol. 2009;10(2):125–34. 10.1016/S1470-2045(08)70339-5 PMC2707020 19138562

[84] Jain N, Roberts KG, Jabbour E, Patel K, Eterovic AK, Chen K (2017). Ph-like acute lymphoblastic leukemia: a high-risk subtype in adults. Blood..

[85] Roberts KG, Gu Z, Payne-Turner D, McCastlain K, Harvey RC, Chen IM, et al. High frequency and poor outcome of Philadelphia chromosome-like acute lymphoblastic leukemia in adults. J Clin Oncol. 2017;35(4):394–401. 10.1200/JCO.2016.69.0073 PMC5455698 27870571

[86] Mullighan CG, Miller CB, Radtke I, Phillips LA, Dalton J, Ma J (2008). BCR-ABL1 lymphoblastic leukaemia is characterized by the deletion of Ikaros. Nature..

[87] Tran TH, Loh ML. Ph-like acute lymphoblastic leukemia. Hematology Am Soc Hematol Educ Program. 2016;2016(1):561–6. 10.1182/asheducation-2016.1.561 PMC6142516 27913529

[88] Roberts KG, Li Y, Payne-Turner D, Harvey RC, Yang YL, Pei D (2014). Targetable kinase-activating lesions in Ph-like acute lymphoblastic leukemia. N Engl J Med.

[89] Jain N, Lamb AV, O’Brien S, Ravandi F, Konopleva M, Jabbour E (2016). Early T-cell precursor acute lymphoblastic leukemia/lymphoma (ETP-ALL/LBL) in adolescents and adults: a high-risk subtype. Blood..

[90] Tasian SK, Loh ML, Hunger SP (2017). Philadelphia chromosome–like acute lymphoblastic leukemia. Blood..

[91] Kim S-K, Knight DA, Jones LR, Vervoort S, Ng AP, Seymour JF (2018). JAK2 is dispensable for maintenance of JAK2 mutant B-cell acute lymphoblastic leukemias. Genes Dev.

[92] DeAngelo DJ, Yu D, Johnson JL, Coutre SE, Stone RM, Stopeck AT (2007). Nelarabine induces complete remissions in adults with relapsed or refractory T-lineage acute lymphoblastic leukemia or lymphoblastic lymphoma: cancer and leukemia group B study 19801. Blood..

[93] Gokbuget N, Basara N, Baurmann H, Beck J, Bruggemann M, Diedrich H (2011). High single-drug activity of nelarabine in relapsed T-lymphoblastic leukemia/lymphoma offers curative option with subsequent stem cell transplantation. Blood..

[94] Berg SL, Blaney SM, Devidas M, Lampkin TA, Murgo A, Bernstein M, et al. Phase II study of nelarabine (compound 506 U78) in children and young adults with refractory T-cell malignancies: a report from the children’s oncology group. J Clinical Oncol. 2005;23(15):3376–82. 10.1200/JCO.2005.03.426 15908649

[95] Dunsmore KP, Winter S, Devidas M, Wood BL, Esiashvili N, Eisenberg N, et al. COG AALL0434: a randomized trial testing nelarabine in newly diagnosed T-cell malignancy. J Clin Oncol. 2018;36(15_suppl):10500. 10.1200/JCO.20.00256 PMC7526719 32813610

[96] Dunsmore KP, Devidas M, Linda SB, Borowitz MJ, Winick N, Hunger SP, et al. Pilot study of nelarabine in combination with intensive chemotherapy in high-risk T-cell acute lymphoblastic leukemia: a report from the children’s oncology group. J Clin Oncol. 2012;30(22):2753–9. 10.1200/JCO.2011.40.8724 PMC3402886 22734022

[97] Abaza Y, Kantarjian HM, Faderl S, Jabbour E, Jain N, Thomas D (2018). Hyper-CVAD plus nelarabine in newly diagnosed adult T-cell acute lymphoblastic leukemia and T-lymphoblastic lymphoma. Am J Hematol.

[98] Coustan-Smith E, Mullighan CG, Onciu M, Behm FG, Raimondi SC, Pei D, et al. Early T-cell precursor leukaemia: a subtype of very high-risk acute lymphoblastic leukaemia. Lancet Oncol. 2009;10(2):147–56. 10.1016/S1470-2045(08)70314-0 PMC2840241 19147408

[99] Neumann M, Heesch S, Schlee C, Schwartz S, Gokbuget N, Hoelzer D (2013). Whole-exome sequencing in adult ETP-ALL reveals a high rate of DNMT3A mutations. Blood..

[100] Zhang J, Ding L, Holmfeldt L, Wu G, Heatley SL, Payne-Turner D (2012). The genetic basis of early T-cell precursor acute lymphoblastic leukaemia. Nature..

[101] Bond J, Graux C, Lhermitte L, Lara D, Cluzeau T, Leguay T, et al. Early response-based therapy stratification improves survival in adult early thymic precursor acute lymphoblastic leukemia: a group for research on adult acute lymphoblastic leukemia study. J Clin Oncol. 2017;35(23):2683–91. 10.1200/JCO.2016.71.8585 28605290

[102] Chonghaile TN, Roderick JE, Glenfield C, Ryan J, Sallan SE, Silverman LB, et al. Maturation stage of T-cell acute lymphoblastic leukemia determines BCL-2 versus BCL-XL dependence and sensitivity to ABT-199. Cancer Discov. 2014;4(9):1074–87. 10.1158/2159-8290.CD-14-0353 PMC4154982 24994123

[103] Jain N, Stevenson KE, Winer ES, Garcia JS, Stone RM, Jabbour E, et al. A multicenter phase I study combining venetoclax with mini-hyper-CVD in older adults with untreated and relapsed/refractory acute lymphoblastic leukemia. Blood. 2019;134(Supplement_1):3867.

[104] Malard F, Mohty M (2020). Acute lymphoblastic leukaemia. Lancet..

[105] DeAngelo DJ, Stevenson KE, Dahlberg SE, Silverman LB, Couban S, Supko JG (2015). Long-term outcome of a pediatric-inspired regimen used for adults aged 18-50 years with newly diagnosed acute lymphoblastic leukemia. Leukemia..

[106] Gökbuget N, Beck J, Brandt K, Brüggemann M, Burmeister T, Diedrich H (2013). Significant improvement of outcome in adolescents and young adults (AYAs) aged 15-35 years with acute lymphoblastic leukemia (ALL) with a pediatric derived adult ALL protocol; results of 1529 AYAs in 2 consecutive trials of the German multicenter study group for adult ALL (GMALL). Blood..

[107] Hayakawa F, Sakura T, Yujiri T, Kondo E, Fujimaki K, Sasaki O, et al. Markedly improved outcomes and acceptable toxicity in adolescents and young adults with acute lymphoblastic leukemia following treatment with a pediatric protocol: a phase II study by the Japan Adult Leukemia Study Group. Blood Cancer J. 2014;4:e252. 10.1038/bcj.2014.72 PMC4220650 25325302

[108] Hough R, Rowntree C, Goulden N, Mitchell C, Moorman A, Wade R (2016). Efficacy and toxicity of a paediatric protocol in teenagers and young adults with Philadelphia chromosome negative acute lymphoblastic leukaemia: results from UKALL 2003. Br J Haematol.

[109] Huguet F, Leguay T, Raffoux E, Thomas X, Beldjord K, Delabesse E, et al. Pediatric-inspired therapy in adults with Philadelphia chromosome-negative acute lymphoblastic leukemia: the GRAALL-2003 study. J Clin Oncol. 2009;27(6):911–8. 10.1200/JCO.2008.18.6916 19124805

[110] Ribera JM, Oriol A, Sanz MA, Tormo M, Fernandez-Abellan P, del Potro E, et al. Comparison of the results of the treatment of adolescents and young adults with standard-risk acute lymphoblastic leukemia with the Programa Espanol de Tratamiento en Hematologia pediatric-based protocol ALL-96. J Clin Oncol. 2008;26(11):1843–9. 10.1200/JCO.2007.13.7265 18398150

[111] Storring JM, Minden MD, Kao S, Gupta V, Schuh AC, Schimmer AD (2009). Treatment of adults with BCR-ABL negative acute lymphoblastic leukaemia with a modified paediatric regimen. Br J Haematol.

[112] Toft N, Birgens H, Abrahamsson J, Griskevicius L, Hallbook H, Heyman M (2018). Results of NOPHO ALL2008 treatment for patients aged 1-45 years with acute lymphoblastic leukemia. Leukemia..

[113] Stock W, Luger SM, Advani AS, Yin J, Harvey RC, Mullighan CG (2019). A pediatric regimen for older adolescents and young adults with acute lymphoblastic leukemia: results of CALGB 10403. Blood..

[114] Rytting ME, Thomas DA, O’Brien SM, Ravandi-Kashani F, Jabbour EJ, Franklin AR (2014). Augmented Berlin-Frankfurt-Munster therapy in adolescents and young adults (AYAs) with acute lymphoblastic leukemia (ALL). Cancer..

[115] Jabbour E, O'Brien S, Ravandi F, Kantarjian H (2015). Monoclonal antibodies in acute lymphoblastic leukemia. Blood..

[116] Hoelzer D, Walewski J, Dohner H, Viardot A, Hiddemann W, Spiekermann K (2014). Improved outcome of adult Burkitt lymphoma/leukemia with rituximab and chemotherapy: report of a large prospective multicenter trial. Blood..

[117] Maury S, Chevret S, Thomas X, Heim D, Leguay T, Huguet F (2016). Rituximab in B-lineage adult acute lymphoblastic leukemia. N Engl J Med.

[118] Thomas DA, O’Brien S, Faderl S, Garcia-Manero G, Ferrajoli A, Wierda W, et al. Chemoimmunotherapy with a modified hyper-CVAD and rituximab regimen improves outcome in de novo Philadelphia chromosome-negative precursor B-lineage acute lymphoblastic leukemia. J Clin Oncol. 2010;28(24):3880–9. 10.1200/JCO.2009.26.9456 PMC2940403 20660823

[119] Thomas DA, Faderl S, O'Brien S, Bueso-Ramos C, Cortes J, Garcia-Manero G (2006). Chemoimmunotherapy with hyper-CVAD plus rituximab for the treatment of adult Burkitt and Burkitt-type lymphoma or acute lymphoblastic leukemia. Cancer..

[120] Rizzieri DA, Johnson JL, Byrd JC, Lozanski G, Blum KA, Powell BL (2014). Improved efficacy using rituximab and brief duration, high intensity chemotherapy with filgrastim support for Burkitt or aggressive lymphomas: cancer and leukemia group B study 10 002. Br J Haematol.

[121] Maloney DG (2012). Anti-CD20 antibody therapy for B-cell lymphomas. N Engl J Med.

[122] Bazarbachi AH, Yilmaz M, Ravandi F, Thomas DA, Khouri M, Garcia-Manero G, et al. A phase 2 study of hyper-CVAD plus ofatumumab as frontline therapy in CD20+ acute lymphoblastic leukemia (ALL): updated results. J Clin Oncol. 2018;36(15_suppl):7041.

[123] Gökbuget N, Hoelzer D. Meningeosis leukaemica in adult acute lymphoblastic leukaemia. J Neurooncol. 1998;38(2):167–80. 10.1023/a:1005963732481 9696368

[124] Lazarus HM, Richards SM, Chopra R, Litzow MR, Burnett AK, Wiernik PH (2006). Central nervous system involvement in adult acute lymphoblastic leukemia at diagnosis: results from the international ALL trial MRC UKALL XII/ECOG E2993. Blood..

[125] Reman O, Pigneux A, Huguet F, Vey N, Delannoy A, Fegueux N (2008). Central nervous system involvement in adult acute lymphoblastic leukemia at diagnosis and/or at first relapse: results from the GET-LALA group. Leuk Res.

[126] Omura GA, Moffitt S, Vogler WR, Salter MM (1980). Combination chemotherapy of adult acute lymphoblastic leukemia with randomized central nervous system prophylaxis. Blood..

[127] Sancho JM, Ribera JM, Oriol A, Hernandez-Rivas JM, Rivas C, Bethencourt C (2006). Central nervous system recurrence in adult patients with acute lymphoblastic leukemia: frequency and prognosis in 467 patients without cranial irradiation for prophylaxis. Cancer..

[128] Pui C-H. Central nervous system disease in acute lymphoblastic leukemia: prophylaxis and treatment. Hematology Am Soc Hematol Educ Program. 2006;2006(1):142–6. 10.1182/asheducation-2006.1.142 17124053

[129] Vora A, Andreano A, Pui CH, Hunger SP, Schrappe M, Moericke A, et al. Influence of cranial radiotherapy on outcome in children with acute lymphoblastic leukemia treated with contemporary therapy. J Clin Oncol. 2016;34(9):919–26. 10.1200/JCO.2015.64.2850 PMC4871998 26755523

[130] Larson RA (2018). Managing CNS disease in adults with acute lymphoblastic leukemia. Leuk Lymphoma.

[131] Jabbour E, Thomas D, Cortes J, Kantarjian HM, O’Brien S (2010). Central nervous system prophylaxis in adults with acute lymphoblastic leukemia: current and emerging therapies. Cancer..

[132] Patel B, Rai L, Buck G, Richards SM, Mortuza Y, Mitchell W (2010). Minimal residual disease is a significant predictor of treatment failure in non T-lineage adult acute lymphoblastic leukaemia: final results of the international trial UKALL XII/ECOG2993. Br J Haematol.

[133] Gokbuget N, Kneba M, Raff T, Trautmann H, Bartram CR, Arnold R (2012). Adult patients with acute lymphoblastic leukemia and molecular failure display a poor prognosis and are candidates for stem cell transplantation and targeted therapies. Blood..

[134] Beldjord K, Chevret S, Asnafi V, Huguet F, Boulland M-L, Leguay T (2014). Oncogenetics and minimal residual disease are independent outcome predictors in adult patients with acute lymphoblastic leukemia. Blood..

[135] Dhedin N, Huynh A, Maury S, Tabrizi R, Beldjord K, Asnafi V, et al. Role of allogeneic stem cell transplantation in adult patients with Ph-negative acute lymphoblastic leukemia. Blood. 2015;125(16):2486–586. 10.1182/blood-2014-09-599894 25587040

[136] Bassan R, Masciulli A, Intermesoli T, Spinelli O, Tosi M, Pavoni C (2016). Final results of Northern Italy leukemia group (NILG) trial 10/07 combining pediatric-type therapy with minimal residual disease study and risk-oriented hematopoietic cell transplantation in adult acute lymphoblastic leukemia (ALL). Blood..

[137] Della Starza I, Chiaretti S, De Propris MS, Elia L, Cavalli M, De Novi LA (2019). Minimal residual disease in acute lymphoblastic leukemia: technical and clinical advances. Front Oncol.

[138] Brüggemann M, Kotrova M. Minimal residual disease in adult ALL: technical aspects and implications for correct clinical interpretation. Blood Adv. 2017;1(25):2456–66. 10.1182/bloodadvances.2017009845 PMC5729622 29296895

[139] Wood B, Wu D, Crossley B, Dai Y, Williamson D, Gawad C (2018). Measurable residual disease detection by high-throughput sequencing improves risk stratification for pediatric B-ALL. Blood..

[140] Short NJ, Jabbour E, Albitar M, de Lima M, Gore L, Jorgensen J (2019). Recommendations for the assessment and management of measurable residual disease in adults with acute lymphoblastic leukemia: a consensus of North American experts. Am J Hematol.

[141] Yilmaz M, Kantarjian H, Wang X, Khoury JD, Ravandi F, Jorgensen J (2020). The early achievement of measurable residual disease negativity in the treatment of adults with Philadelphia-negative B-cell acute lymphoblastic leukemia is a strong predictor for survival. Am J Hematol.

[142] Terwey TH, Hemmati PG, Nagy M, Pfeifer H, Gokbuget N, Bruggemann M, et al. Comparison of chimerism and minimal residual disease monitoring for relapse prediction after allogeneic stem cell transplantation for adult acute lymphoblastic leukemia. Biol Blood Marrow Transplant. 2014;20(10):1522–9. 10.1016/j.bbmt.2014.05.026 24907626

[143] Zhao XS, Liu YR, Zhu HH, Xu LP, Liu DH, Liu KY (2012). Monitoring MRD with flow cytometry: an effective method to predict relapse for ALL patients after allogeneic hematopoietic stem cell transplantation. Ann Hematol.

[144] Giebel S, Stella-Holowiecka B, Krawczyk-Kulis M, Gökbuget N, Hoelzer D, Doubek M (2010). Status of minimal residual disease determines outcome of autologous hematopoietic SCT in adult ALL. Bone Marrow Transplant.

[145] FDA expands approval of Blincyto for treatment of a type of leukemia in patients who have a certain risk factor for relapse [Available from: https://www.fda.gov/news-events/press-announcements/fda-expands-approval-blincyto-treatment-type-leukemia-patients-who-have-certain-risk-factor-relapse. Accessed 30 Apr 2020.

[146] Giebel S, Marks DI, Boissel N, Baron F, Chiaretti S, Ciceri F (2019). Hematopoietic stem cell transplantation for adults with Philadelphia chromosome-negative acute lymphoblastic leukemia in first remission: a position statement of the European working group for adult acute lymphoblastic leukemia (EWALL) and the acute leukemia working party of the European Society for Blood and Marrow Transplantation (EBMT). Bone Marrow Transplant.

[147] Issa GC, Kantarjian HM, Yin CC, Qiao W, Ravandi F, Thomas D (2017). Prognostic impact of pretreatment cytogenetics in adult Philadelphia chromosome-negative acute lymphoblastic leukemia in the era of minimal residual disease. Cancer..

[148] Trinquand A, Tanguy-Schmidt A, Ben Abdelali R, Lambert J, Beldjord K, Lengline E, et al. Toward a NOTCH1/FBXW7/RAS/PTEN-based oncogenetic risk classification of adult T-cell acute lymphoblastic leukemia: a group for research in adult acute lymphoblastic leukemia study. J Clin Oncol. 2013;31(34):4333–42. 10.1200/JCO.2012.48.5292 24166518

[149] Hoelzer D, Gokbuget N (2009). T-cell lymphoblastic lymphoma and T-cell acute lymphoblastic leukemia: a separate entity?. Clin Lymphoma Myeloma.

[150] Pehlivan KC, Duncan BB, Lee DW. CAR-T cell therapy for acute lymphoblastic leukemia: transforming the treatment of relapsed and refractory disease. Curr Hematol Malig Rep. 2018;13(5):396–406. 10.1007/s11899-018-0470-x 30120708

[151] Srour SA, Milton DR, Bashey A, Karduss-Urueta A, Al Malki MM, Romee R, et al. Haploidentical transplantation with post-transplantation cyclophosphamide for high-risk acute lymphoblastic leukemia. Biol Blood Marrow transplant. 2017;23(2):318–24. 10.1016/j.bbmt.2016.11.008 PMC5517662 27856368

[152] Brissot E, Labopin M, Russo D, Martin S, Schmid C, Glass B, et al. Alternative donors provide comparable results to matched unrelated donors in patients with acute lymphoblastic leukemia undergoing allogeneic stem cell transplantation in second complete remission: a report from the EBMT Acute Leukemia Working Party. Bone Marrow Transplant. 2020. 10.1038/s41409-020-0849-x 32203261

[153] Wang Y, Liu QF, Xu LP, Liu KY, Zhang XH, Ma X, et al. Haploidentical versus matched-sibling transplant in adults with Philadelphia-negative high-risk acute lymphoblastic leukemia: a biologically phase III randomized study. Clin Cancer Res. 2016;22(14):3467–76. 10.1158/1078-0432.CCR-15-2335 26927664

[154] Chang YJ, Wang Y, Xu LP, Zhang XH, Chen H, Chen YH (2020). Haploidentical donor is preferred over matched sibling donor for pre-transplantation MRD positive ALL: a phase 3 genetically randomized study. J Hematol Oncol.

[155] Rosko A, Wang HL, de Lima M, Sandmaier B, Khoury HJ, Artz A (2017). Reduced intensity conditioned allograft yields favorable survival for older adults with B-cell acute lymphoblastic leukemia. Am J Hematol.

[156] Aldoss I, Forman SJ, Pullarkat V. Acute lymphoblastic leukemia in the older adult. J Oncol Pract. 2019;15(2):67–75. 10.1200/JOP.18.00271 30763199

[157] Roth-Guepin G, Canaani J, Ruggeri A, Labopin M, Finke J, Cornelissen JJ (2017). Allogeneic stem cell transplantation in acute lymphoblastic leukemia patients older than 60 years: a survey from the acute leukemia working party of EBMT. Oncotarget..

[158] Qasim W, Allogeneic CAR (2019). T cell therapies for leukemia. Am J Hematol.

[159] Fry TJ, Shah NN, Orentas RJ, Stetler-Stevenson M, Yuan CM, Ramakrishna S (2018). CD22-targeted CAR T cells induce remission in B-ALL that is naive or resistant to CD19-targeted CAR immunotherapy. Nat Med.

[160] Amrolia PJ, Wynn R, Hough RE, Vora A, Bonney D, Veys P, et al. Phase I study of AUTO3, a bicistronic chimeric antigen receptor (CAR) T-cell therapy targeting CD19 and CD22, in pediatric patients with relapsed/refractory B-cell acute lymphoblastic leukemia (r/r B-ALL): Amelia Study. Blood. 2019;134(Supplement_1):2620.

[161] Schultz LM, Muffly LS, Spiegel JY, Ramakrishna S, Hossain N, Baggott C, et al. Phase I trial using CD19/CD22 bispecific CAR T cells in pediatric and adult acute lymphoblastic leukemia (ALL). Blood. 2019;134(Supplement_1):744.

[162] Liu E, Marin D, Banerjee P, Macapinlac HA, Thompson P, Basar R (2020). Use of CAR-transduced natural killer cells in CD19-positive lymphoid tumors. N Engl J Med.

[163] Bride KL, Vincent TL, Im SY, Aplenc R, Barrett DM, Carroll WL, et al. Preclinical efficacy of daratumumab in T-cell acute lymphoblastic leukemia. Blood. 2018;131(9):995-9. 10.1182/blood-2017-07-794214 PMC5833263 29305553

[164] Ganzel C, Kharit M, Duksin C, Rowe JM (2018). Daratumumab for relapsed/refractory Philadelphia-positive acute lymphoblastic leukemia. Haematologica..

[165] Cooper ML, Choi J, Staser K, Ritchey JK, Devenport JM, Eckardt K (2018). An “off-the-shelf” fratricide-resistant CAR-T for the treatment of T cell hematologic malignancies. Leukemia..

[166] Wang SL X, Gao L, Yuan Z, Wu K, Liu L, Luo L, et al. Clinical safety and efficacy study of TruUCAR™ GC027: the first-in-human, universal CAR-T therapy for adult relapsed/refractory T-cell acute lymphoblastic leukemia (r/r T-ALL). American Association for Cancer Research (AACR) Virtual Annual Meeting I; April 27-28, 2020.

